# Investigating neural impairments in psychotic disorders using electroencephalography and cortical spheroids

**DOI:** 10.1038/s41398-026-03863-4

**Published:** 2026-02-17

**Authors:** Denis Reis de Assis, Atle Bråthen Pentz, Jordi Requena Osete, Oleksandr Ievglevskyi, Matthieu Vandenberghe, Ibrahim Ahmed Akkouh, Tuomo Mäki-Marttunen, Erik G. Jönsson, Ole A. Andreassen, Srdjan Djurovic, Elena Kondratskaya, Torbjørn Elvsåshagen

**Affiliations:** 1 Center for Precision Psychiatry Division of Mental Health and Addiction, Oslo University Hospital & Institute of Clinical Medicine, University of Oslo, and Division of Mental Health and Addiction, Oslo University Hospital, Oslo, Norway; 2 Department of Medical Genetics, Oslo University Hospital, Oslo, Norway; 3 Faculty of Medicine and Health Technology, Tampere University, Tampere, Finland; 4 Department of Biosciences, University of Oslo, Oslo, Norway; 5 Centre for Psychiatric Research, Department of Clinical Neuroscience, Karolinska Institutet & Stockholm Health Care Sciences, Stockholm Region, Stockholm, Sweden; 6 Department of Neurology, Oslo University Hospital, Oslo, Norway; 7 Department of Behavioural Medicine, Institute of Basic Medical Sciences, University of Oslo, Oslo, Norway

**Keywords:** Bipolar disorder, Schizophrenia, Stem cells, Molecular neuroscience

## Abstract

Synaptic dysfunction is a candidate mechanism in psychotic disorders, yet the precise underlying substrates remain elusive. We investigated how combining in vivo electroencephalography (EEG) and in vitro human cortical spheroid (hCS)-based methods can further our understanding of psychosis pathophysiology during fetal stages of neurodevelopment. Ten individuals with schizophrenia (SZ) or bipolar disorder (BD; 5 males and 5 females) and five controls (CTRL; 3 males and 2 females) underwent EEG assessments, including long-term potentiation (LTP)-like cortical plasticity and mismatch negativity (MMN). hCS were generated from induced pluripotent stem cells of all participants, and immunohistochemistry, Seahorse bioenergetics and patch-clamp recordings were performed. EEG-based LTP-like plasticity was reduced in individuals with SZ and BD. Basal respiration was decreased in BD hCS and VGLUT1 levels were reduced in both SZ and BD hCS. There was a positive association between EEG-based LTP-like plasticity and hCS basal respiration which survived correction. Our data provide further support for roles of mitochondrial and glutamatergic impairments in the synaptic dysfunction of psychosis and demonstrate the potential of combining EEG- and hCS-based methods for early development mechanistic studies of brain disorders.

## Introduction

Schizophrenia (SZ) and bipolar disorder (BD) are severe mental illnesses affecting ~3% of the population [1, 2]. The disorders are among the leading causes of disability worldwide and have overlapping clinical presentations and genetic architectures [3, 4]. While the precise neural underpinnings of SZ and BD remain to be clarified, recent genetic findings suggest synaptic dysfunction as a central mechanism [5, 6]. There is, however, limited in vivo evidence for cerebral synaptic impairments in SZ and BD due to a lack of methods for direct investigations of synaptic function in humans.

Electroencephalography (EEG) enables indirect measurements of postsynaptic potentials in the cerebral cortex [7]. EEG-based methods are available for studies of synaptic transmission and plasticity in the cortex, including long-term potentiation (LTP)-like cortical plasticity and mismatch negativity (MMN). The LTP-like cortical plasticity assay shares features with canonical glutamatergic LTP [8–10] and is reduced in individuals with SZ or BD [8, 11, 12]. MMN is a negative EEG wave typically elicited by the presentation of a deviant stimulus within a stream of familiar events [13], depends on intact neurotransmission through glutamatergic and GABAergic synapses [14, 15], and is impaired in SZ and BD [16–19]. Thus, results from LTP-like cortical plasticity and MMN experiments are consistent with glutamatergic and GABAergic synaptic dysfunction in SZ and BD, yet the exact underlying mechanisms are unknown.

Synaptic activity is a highly energy demanding process, in which neurons consume between 75–80% of the brain energy, especially on ionic pumps to reestablish electrochemical gradients dissipated by postsynaptic potentials and presynaptic vesicle recycling [20]. Indeed, a tight association between energy demand and synaptic activity is evidenced by a spatial coincidence between functional magnetic resonance imaging responses and EEG-recorded local field potentials [21], indicating the synapse as the major energy demanding site of the nervous system [22]. Importantly, bioenergetics status has a tight influence especially on brain glutamatergic synapse [22, 23].

Mitochondrial dysfunction has been reported in SZ and BD patients [24, 25], and considered a candidate cause of disturbed neural plasticity in both disorders [26, 27]. This has been supported by genetic [28] and functional enrichment analysis involving genes implicated in mitochondrial function [29]. In addition, defects in synaptic plasticity and energy metabolism have been common findings in both SZ and BD [30]. However, the absence of suitable disease models to access live human brain tissue has hampered more detailed studies about the involved mechanisms, and on the association between synaptic activity and bioenergetics in these disorders.

Induced pluripotent stem cell (iPSC)-derived three dimensional neural tissues have emerged as promising models for mechanistic studies of human disease [31, 32]. Particularly, human iPSC-derived cortical spheroids (hCS) recapitulate early cortical development and enable investigations of synaptic structure and transmission in vitro [33, 34]. Recent hCS studies in SZ and BD have found aberrant neuronal excitability, neurite outgrowth, neurogenesis [35–40], and brain energy metabolism [37, 39, 41].

In the current study, we combined in vivo EEG and in vitro hCS methods to further our understanding of neural dysfunction in SZ and BD. First, individuals with SZ or BD and healthy controls underwent EEG-based assessments of synaptic function as indexed by MMN and LTP-like cortical plasticity. In parallel, we generated hCS from these participants and assessed the structure and function of the hCS.

We hypothesize that impaired MMN and LTP-like plasticity in SZ and BD versus controls are reflected in the in vitro hCS which will exhibit abberrant neurotransmission, electrophysiological and energy metabolism phenotypes associated with in vivo MMN and LTP-like plasticity.

## Methods

### Participants and clinical assessments

Seven females and eight males with a mean age of 42.8 years (range, 23-58) were included. No significant group differences were found regarding sex, age, or IQ (Supplementary Table 1). The patients were recruited through psychiatric in- and outpatient units in the greater Oslo area, while healthy controls (CTRL) were recruited through national records as previously detailed [42]. The participants were diagnosed according to the Diagnostic and Satistical Manual of Mental Disorders, Fourth Edition (DSM-IV) criteria and submitted to standardized clinical evaluation as described in previous work [43] and in Supplementary Material. All participants had normal or corrected-to-normal vision and a hearing threshold of < 40 dB. The study was approved by the Norwegian Data Protection Agency and the Regional Committee for Medical Research Ethics South East Norway and all participants provided informed consent. All methods were performed in accordance with the ethical standards of the aforementioned committee and with the 1964 Helsinki Declaration and its later amendments.

### EEG acquisition and preprocessing

EEG acquisition and processing are detailed in the Supplementary Material. In brief, scalp EEG data from the LTP-like cortical plasticity and MMN paradigms was amplified (-3dB at 417 Hz low-pass, DC-coupled) and digitized (2048 Hz) from 72 Ag/AgCl active electrodes using a Biosemi Active-Two amplifier (BioSemi, Amsterdam, The Netherlands). The offline preprocessing of EEG data was conducted in Matlab 2017a (Mathworks Inc., Natick, MA) using EEGLAB [44] and in-house scripts. Here, the data was low-pass filtered, downsampled, high-pass filtered, and processed using the PREP pipeline [45]. Independent component analysis (ICA) was conducted and components representing artefacts were identified using ICLabel [46] and removed. Epochs were baseline-corrected and those with amplitudes exceeding ±100 mV were rejected.

### LTP-like cortical plasticity and mismatch negativity paradigms

The LTP-like cortical plasticity paradigm was performed as previously described [12, 47]. Visual evoked potentials (VEPs) were evoked by checkerboard reversals presented on a 24-inch 144 Hz AOC LCD screen in two premodulation blocks before and eight blocks after a plasticity-inducing intervention block. All baseline and postintervention blocks lasted ~40 s, while the stimulation block lasted 10 min. Postintervention blocks were presented at 2 min, 3 min 40 s, 6 min 20 s, 8 min, ~30 min, ~32 min, ~54 min, and ~56 min after the intervention block. Since the baseline consisted of two VEP blocks, postintervention blocks were also combined into series of two blocks for equal comparison, resulting in one baseline assessment and four postintervention assessments. LTP-like cortical plasticity was defined as the N1b amplitude change from baseline to the four postintervention blocks. The N1b change is a robust correlate of LTP-like cortical plasticity, which is reduced in SZ and BD [8, 12, 47].

MMN was obtained from the FCz electrode using a roving paradigm [48] scripted and presented with PsychToolbox in Matlab. Trains of identical standard auditory stimuli with regards to pitch and duration were presented binaurally by earphones (ER-2, Etymotic Research, Inc., Elk Grove Village, IL, USA) in a pseudorandom fashion with 2, 6, or 18 repetitions alternating with a new train of stimuli with different physical properties, thus resulting in the first tone of a new train acting as a deviant stimulus relative to last tone of the previous stimulus train [49]. A grand average MMN (hereafter referred to as “MMN”) was estimated by subtracting the amplitude of the deviant waveforms from that of the waveforms elicited by the preceding standard stimuli.

### IPSC derivation and maintenance

Skin fibroblasts obtained from the participants were grown in DMEM supplemented with 10% fetal bovine serum, 1% penicillin streptomycin, and 1% Glutamax and reprogrammed with Sendai virus, transduced with the CytoTune™-iPS 2.0 Sendai Reprogramming Kit (ThermoFisher) containing Oct4, Sox2, Klf4 and c-Myc reprogramming factors according to the manufacturer. After 7 days cells were plated on Vitronectin and the medium was changed to Essential 8 Flex Medium (ThermoFisher). iPSCs were subjected to phenotyping and monitored for morphology and pluripotency marker expressions at the Norwegian Core Facility for Human Pluripotent Stem Cell Research Centre, as previously detailed [35, 37]. The iPSC were also tested for karyotyping using a KaryoStat GeneChip array (ThermoFisher).

### Cortical spheroids generation

All hCS were generated from iPSCs following the method of Yoon and colleagues [50] in two of our previous studies [35, 37] and examined at an age of 180 days in vitro (DIV) in this study. A detailed protocol can be found in Supplementary Material. Immunohistochemistry, bioenergetics measurements, and patch clamp electrophysiology were performed in those two batches of hCS (CTRL and SZ, and CTRL and BD) [35, 37]. CTRL hCS of both batches were generated from the same five healthy controls included in this study. We also used cell transcriptomic data from these hCS for cell type composition through deconvolution analysis (Supplementary Material).

### hCS electrophysiology

The hCS were cut into slices in standard ASCF for both electrophysiology recordings and Seahorse bioenergetics. Whole-cell patch-clamp technique in slices from one hCS per participant was applied for functional characterization of neurons in the hCS. Passive and active neuronal properties were compared for multiple neuronal characteristics at a single-cell level both in voltage and current clamp recording modes as described in details in the Supplementary Material.

### Cryosectioning and immunohistochemistry

One hCS per participant was fixed in 4% PFA in PBS O/N at 4 °C, transferred to 30% sucrose for 24 h, embedded into OCT compound and stored at -80 °C. The hCS were cut into 10 µm sections using a cryostat for immunohistochemistry analysis. Sections were washed with PBS and blocked in PBS with 6% donkey serum, 0.2% Triton X-100 diluted in PBS for 1 h at room temperature. Sections were incubated with primary antibodies O/N at 4 °C. After washing with PBS, sections were incubated with secondary antibodies for 2 h at 37 °C. Primary and secondary antibodies and dilutions used are specified in the Supplementary Table 2. Nuclei were stained with DAPI and then cover glasses were mounted on top with Fluoromount-G. Images were acquired using a Zeiss LSM 700 confocal microscope and processed using Fiji software. We quantified VGLUT1 and MAP2 fluorescence and calculated Manders’ colocalization coefficient M1 (VGLUT1 signal overlapping MAP2 signal) using Fiji (JACoP). Since M1 did not differ between groups, we estimated VGLUT1 abundance per cell by calculating the % of VGLUT1 positive cells. 300 cells were analysed for each marker from all lines. Additional details are provided in the Supplementary Material.

### Bioenergetics assessment

Bioenergetics parameters were measured in slices from one or two hCS per participant according to our previous report [25]. Slices from hCS were placed in HEPES-based ACSF and allowed to recover at room temperature for 10 min. Then, 5 slices were transferred to each well of XFe24-well plates filled with unbuffered media, and incubated for 1 h in a non-CO2 incubator at 37 °C. First, basal levels of oxygen consumption rate (OCR) and extracellular acidity rate (ECAR) were recorded, using a Seahorse XFe24 extracellular flux analyser (Agilent Technologies, Santa Clara, CA, US). Then the complex V inhibitor oligomycin was added to determine ATP respiration, the uncoupling agent carbonyl cyanide-p-trifluoromethoxyphenylhydrazone (CCCP) was added to quantify maximal respiration, and finally the oxidative phosphorylation blockers rotenone and antimycin A were concomitantly added to determine non-respiratory oxygen consumption.The experiments were run in duplicates and repeated at least twice. The total protein content in each well was measured by the BCA protein assay kit (ThermoFisher #23225) and used to normalize all OCR and ECAR parameters.

### Statistical analysis

To reduce potential bias, investigators were blinded to participants’ identities and group assignments throughout experimental procedures and statistical analyses. Variances were similar between the groups being compared. Group comparisons were performed using parametric tests with a significance threshold of *P* < 0.05, in R version 4.2.2, GraphPad Prism version 9.5.1, p-CLAMP 10, and OriginPro. For group analyses of EEG indices, the CTRL group was compared to the combined patient sample of individuals with SZ or BD. For hCS read-outs, the CTRL group was compared to SZ or to BD in separate analyses and to the combined group of SZ and BD.

We then examined whether group differences in EEG indices could be predicted by hCS variables. As primary analyses we investigated those hCS read-outs differing by groups. We first averaged the results from the two batches in the CTRL group then performed backward stepwise linear regression across the whole sample including batch, diagnosis, age and sex in the initial model. We subsequently ran secondary analyses exploring the associations between both EEG indices and remainder of the hCS read-outs as well as with clinical variables (PANSS score, IQ, GlobaL Assessment of Functioning).

## Results

### Demographics and clinical characteristics

Ten individuals with SZ (3 males and 2 females) or BD (2 males and 3 females) and five CTRL (3 males and 2 females) whose LTP-like plasticity and MMN data were part of published works with larger samples of patients and controls (*N* = 300 and *N* = 400 for LTP-like plasticity and MMN, respectively) [12, 51] provided skin biopsies for the generation of iPSCs and hCS as outlined in previous studies [35, 37]. Demographic and clinical details about the participants are provided in Supplementary Table 1.

### In vivo EEG

**Fig. 1:**
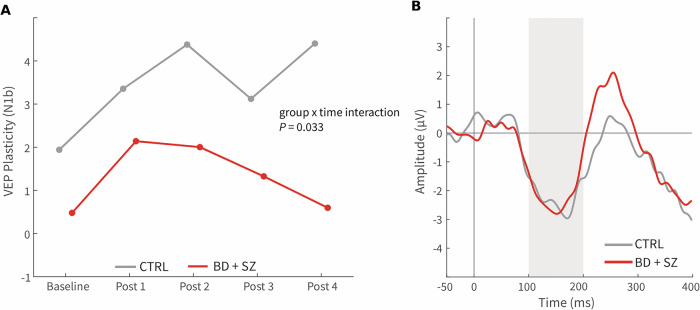
EEG data from the study participants. Event related potentials in CTRL (gray color) and the combined patient group (BD + SZ, red colour) showing (**A**) the amplitude of the N1 b component of the Visual-evoked potential (VEP) at baseline and throughout the four post-modulation blocks showing a group x time interaction suggesting reduced amplitude during the later blocks in the patient group, indicative of reduced long-term potentiation (LTP)-like plasticity in patients. (**B**) Event related potentials of the auditory mismatch negativity (MMN) in patients (BD + SZ) and CTRL showing no difference in mean amplitude in the 100-200 ms post-stimulus interval. CTRL, N = 5; SZ, N = 5; BD, N = 5. Data were analyzed using general linear models.

For LTP-like cortical plasticity, there was a significant group x time interaction (*F* (3,33) = 3.27, *P* = 0.033), thus indicating a greater reduction in plasticity over time in the patients (Fig. 1A). Moreover, there was a trend towards reduced LTP-like plasticity in the patients at the last (the fourth) postintervention assessment (*F* (1,11) = 4.03, *P* = 0.070; Fig. 1A). No significant group difference was found for MMN (*F* (1,13) < 0.01, *P* = 0.97 (Fig. 1B)).

### Cell type composition and electrophysiology characterization of hCS neurons

**Fig. 2:**
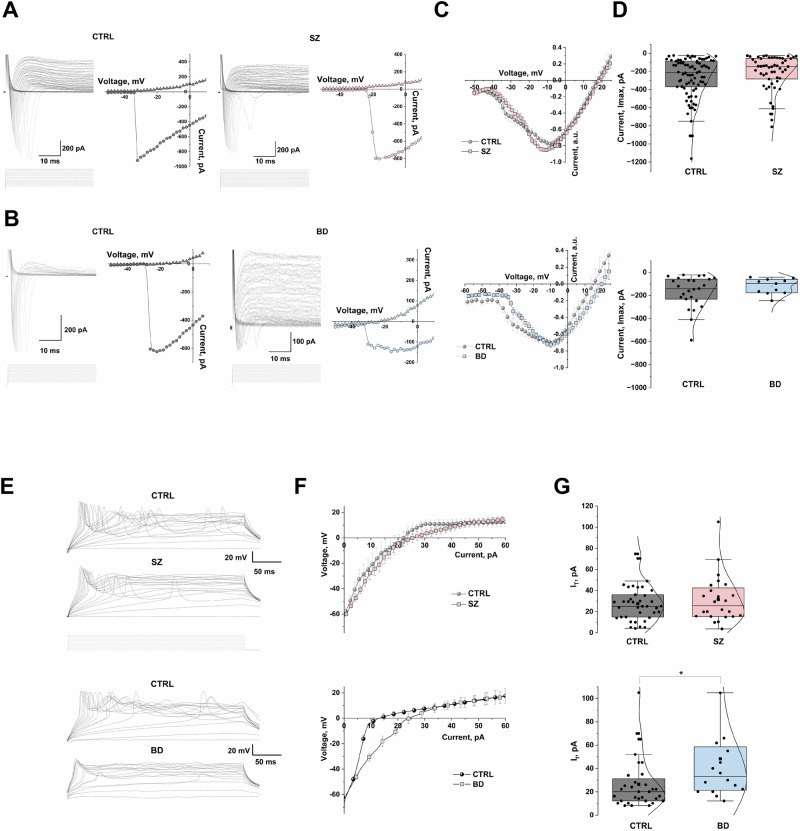
Whole-cell patch-clamp electrophysiology of hCS neurons. (**A, B**) Representative transmembrane current trace recorded from CTRL/SZ, and from CTRL/BD neurons with corresponding sodium current-voltage relation curve (IV. Currents were elicited by stepping the membrane potential from -70 to +40 mV in 2 mV increment as shown in protocol (lower panel). The inward currents were completely and reversibly blocked by TTX (1 mM, data not shown), thus referred to be mediated by TTX-sensitive voltage-gated sodium channels. (**C**) Normalized current-voltage relationship curves of inward sodium currents in all groups, CTRL (N = 33) vs. SZ (N = 27) and CTRL (N = 14) vs. BD group (N = 14) One organoid per line was used for electrophysiological assessment. For group analysis of CTRL vs. SZ, the recordings from 3-6 neurons per line. For comparison of CTRL vs. BD, 2-3 neurons were analysed per line (in total we analyzed data from 89 neurons from all experimental groups). BD group data were analysed from 4 out 5 donors due to unreliable electrophysiological recordings from one of the lines. (**D)** Comparison of Na current max amplitude (I_max_) in CTR vs SZ and CTRL vs BD neuron groups. Averaged mean (I_max_) calculated for CTRL vs SZ and CTRL vs BD were not statistically different. Data analyzed by two sample t test (Box chart represent 25–75% data range, whiskers 5–95% correspondingly; median represented as strait line and mean value as square symbol). Each dot represents an individual recording. (**E**) Representative traces of evoked action potentials (AP), in current clamp mode, recorded from CTRL and SZ individual neurons, and for CTRL and BD neurons. The lower panel under each graph represents the protocol for incremental pulse step applied (2 pA step). (**F**) average VI curves correspond to current injection versus membrane potential changes in all groups. (**G**) Comparison of current threshold (It) for AP induction in CTR vs SZ and CTRL vs BD neuron groups. Each dot represents an individual recording. Averaged (I_t_) calculated for CTRL vs SZ were not different (*P* = 0.862), whereas I_t_ for BD group was significantly higher as compared to age matched CTRL (*P* = 0.036). Data analyzed by two sample t test.

First, we identified cell type composition of hCS by deconvolution analysis of sequenced RNA. As expected, we confirmed that most of the mature neurons in the hCS are glutamatergic and express the glutamatergic neuron marker *SLC17A7* (*VGLUT1*) (Supplementary Figure 1). Then, analyses of passive and active properties of hCS neurons were performed based on electrophsiological recordings from 88 cells, which produced a large transient inward currents and sustained outward currents in response to stepping voltage, typical for neurons. Inward currents (tested in some neurons) were reversibly blocked by TTX (1 µM, data not shown), thus mediated by TTX-sensitive voltage-gated sodium channels. The representative Na^+^, recorded in control and SZ neurons, as well as in control and BD neurons and corresponding IV curves are shown in Fig. 2A, B. The normalized voltage-current relation (IV) curves for CTRL vs SZ and CTRL vs BD are shown in Fig. 2C. BD hCS presented a slightly delayed activation and the curve was shifted to increased depolarizing potentials compared to CTRL hCS (Fig. 2C, lower panel).

There were no significant differences between CTRL and patients in averaged rest membrane potential (Supplementary Figure 2C) or amplitudes of Na^+^ inward currents, two sample t test (Fig. 2D, Supplementary Table 3). Additional membrane parameters are compared in Supplementary Figure 2.

Most neurons reliably produced a single action potential in response to incremental depolarizing current pulses (representative traces from CTRL and SZ and groups CTRL and BD neurons are shown in Fig. 2E, and the corresponding VI curves in Fig. 2F). The average threshold current required to elicit the first action potential (It) did not differ between CTRL and SZ hCS. However, it was significantly higher in BD hCS compared to CTRL hCS (two-sample t-test, *P* = 0.036; Fig. 2G). These findings indicate that BD hCS neurons exhibit reduced excitability relative to controls, which is consistent with our previous study involving a larger cohort of BD samples. This observation also aligns with earlier reports showing a markedly reduced number of regions of interest (ROIs) and diminished calcium transient amplitudes in the BD group [37].

We analysed functional network properties in SZ and CTRL hCS via assessments of spontaneous excitatory postsynaptic currents (sEPSCs). In a voltage clamp mode (Vh = -70 mV), some neurons exhibited sEPSCs with an amplitude range of 2-10 pA (the original current traces shown in the Supplementary Figure 3D-F). Comparison of sEPSCs parameters from SZ hCS vs CTRL showed significantly smaller amplitudes of sEPSCs in the SZ group (two sample t test, *P* = 0.041) (Supplementary Figure 3E, Supplementary Table 4), while frequencies and current kinetic parameters (τ rise and τ decay; Supplementary Figure 3F, Supplementary Table 4) did not present any differences (two sample t test, *P* ≥ *0.05)*.

### Immunohistochemistry characterization

**Fig. 3:**
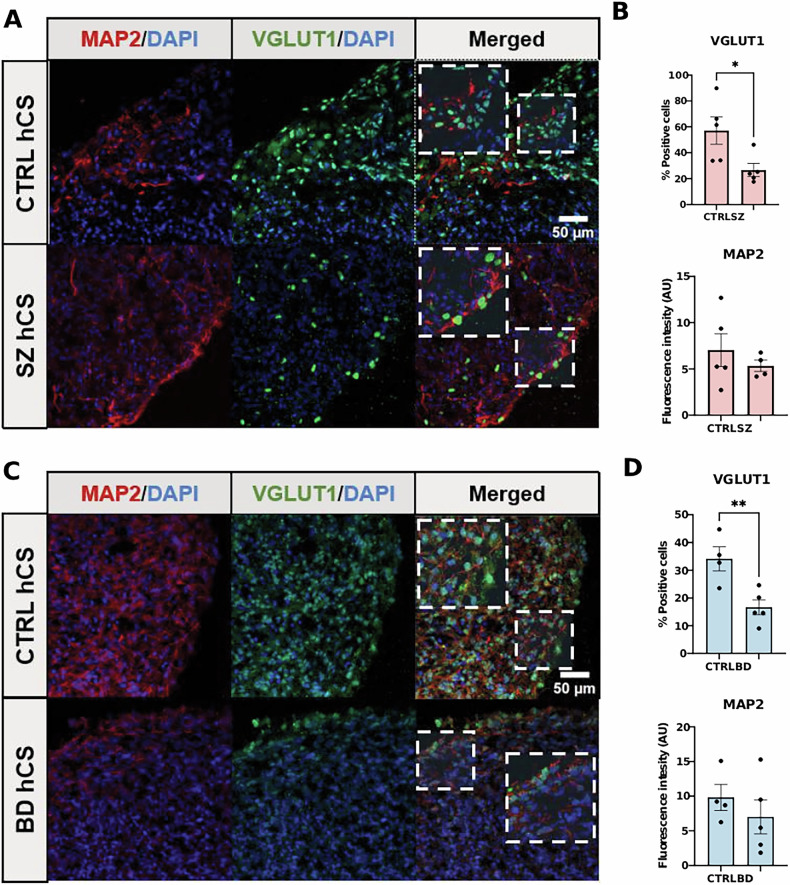
Immunohistochemistry expression of microtubule-associated protein 2 (MAP2) and vesicular glutamate transporter 1 (VGLUT1) in hCS. (**A**) Representative immunofluorescence staining for the neuron marker MAP2 and the vesicular glutamatergic protein VGLUT1 in hCS derived from CTRL and SZ patients. (**B**) MAP2 and VGLUT1 intensity quantification showing VGLUT1 deficit in CTRL and SZ patients. (**C**) Representative immunofluorescence staining for the neuron marker MAP2 and the vesicular glutamatergic protein VGLUT1 in hCS derived from CTRL and BD patients. (**D**) MAP2 and VGLUT1 intensity quantification showing VGLUT1 deficit in CTRL and BD patients. The percentage of cells positive for VGLUT1 for each donor were calculated by counting at least 300 cells from different fields within the cortical plate region of the hCS. Bar graphs with overlaid individual data points. Bars represent the mean ± standard error (SEM) (CTRL, N = 5; SZ, N = 5; BD, N = 5. *P ≤ 0.05, **P ≤ 0.01). Data were analyzed by unpaired Student t test.

We first measured levels of neuronal plasticity markers in the hCS from the patients and CTRL. These included the neuronal marker microtubule-associated protein 2 (MAP2), vesicular glutamate transporter 1 (VGLUT1), which is key to glutamatergic neurotransmission. We were able to identify the expression of VGLUT1 and MAP2 (Fig. 3). There were no differences in Manders’ colocalization coefficient M1 between BD or SZ and CTRL (CTRL vs. SZ: t(8) = 0.524, *P* = 0.614; CTRL vs. BD: t(7) = 1.664, *P* = 0.140), and VGLUT1 levels were reduced in both SZ (*F* (1,7) = 11.66, *P* = 0.011) and BD hCS (*F* (1,7) = 12.84, *P* = 0.009). When rerunning the group analyses for the combined patient sample, VGLUT1 was significantly reduced in the patients relative to CTRL (*F* (1,12) = 17.21, *P* = 0.001). These findings suggest that VGLUT1 reduction, which is key to glutamatergic neurotransmission, may be a trait shared between SZ and BD.

### Bioenergetics assessment of hCS

**Fig. 4:**
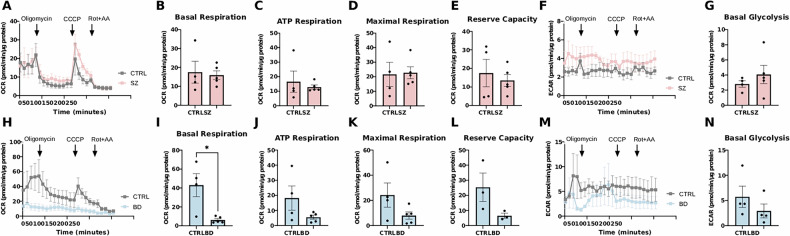
Seahorse bioenergetics in hCS. (**A,** **F**) Representative traces of oxidative phosphorylation rate (OXPHOS) and of extracellular acidification rate (ECAR) recorded from hCS slices in CTRL and SZ hCS, showing no differences in both OXPHOS parameters (**B-E**), and basal glycolysis (**G**) in SZ hCS as compared to CTRL hCS. (**H,** **M**) Representative traces of oxidative phosphorylation rate (OXPHOS) and of extracellular acidification rate (ECAR) recorded from hCS slices in CTRL and BD hCS, showing no differences in ATP respiration, maximal respiration and reserve capacity (**J-L**), decreased basal respiration in BD hCS as compared to CTRL hCS (**I**), and no differences in basal glycolysis in BD hCS as compared to CTRL hCS (**N**). Two organoids per line were analyzed for mitochondrial assessment. All experiments were run in duplicates, and values were normalized to total protein. Bar graphs with overlaid individual data points. Bars represent the mean ± standard error (SEM) CTRL, N = 5; SZ, N = 5; BD, N = 5. *P ≤ 0.05. Data were analyzed by unpaired Student t test.

To examine the bioenergetics phenotype of hCS derived from SZ and BD patients, we measured Seahorse OXPHOS and ECAR parameters (Fig. 4). SZ hCS presented normal OXPHOS kinetic graph (Fig. 4A) and mitochondrial respiration parameters (Fig. 4B-E). Despite there was an apparent increase in the ECAR kinetic graph (Fig. 4F), basal glycolysis (Fig. 4G) was unchanged (*P* = 0.7754). In contrast, BD hCS showed a reduced OXPHOS kinetic graph (Fig. 4H), of which basal respiration was decreased (*P* = 0.0115) (Fig. 4I). BD hCS did not present any changes in the ECAR kinetic graph (Fig. 4M) or in basal glycolysis (Fig. 4N) (*P* = 0.0688).

### Associations between in vivo EEG and in vitro hCS read-outs

**Fig. 5:**
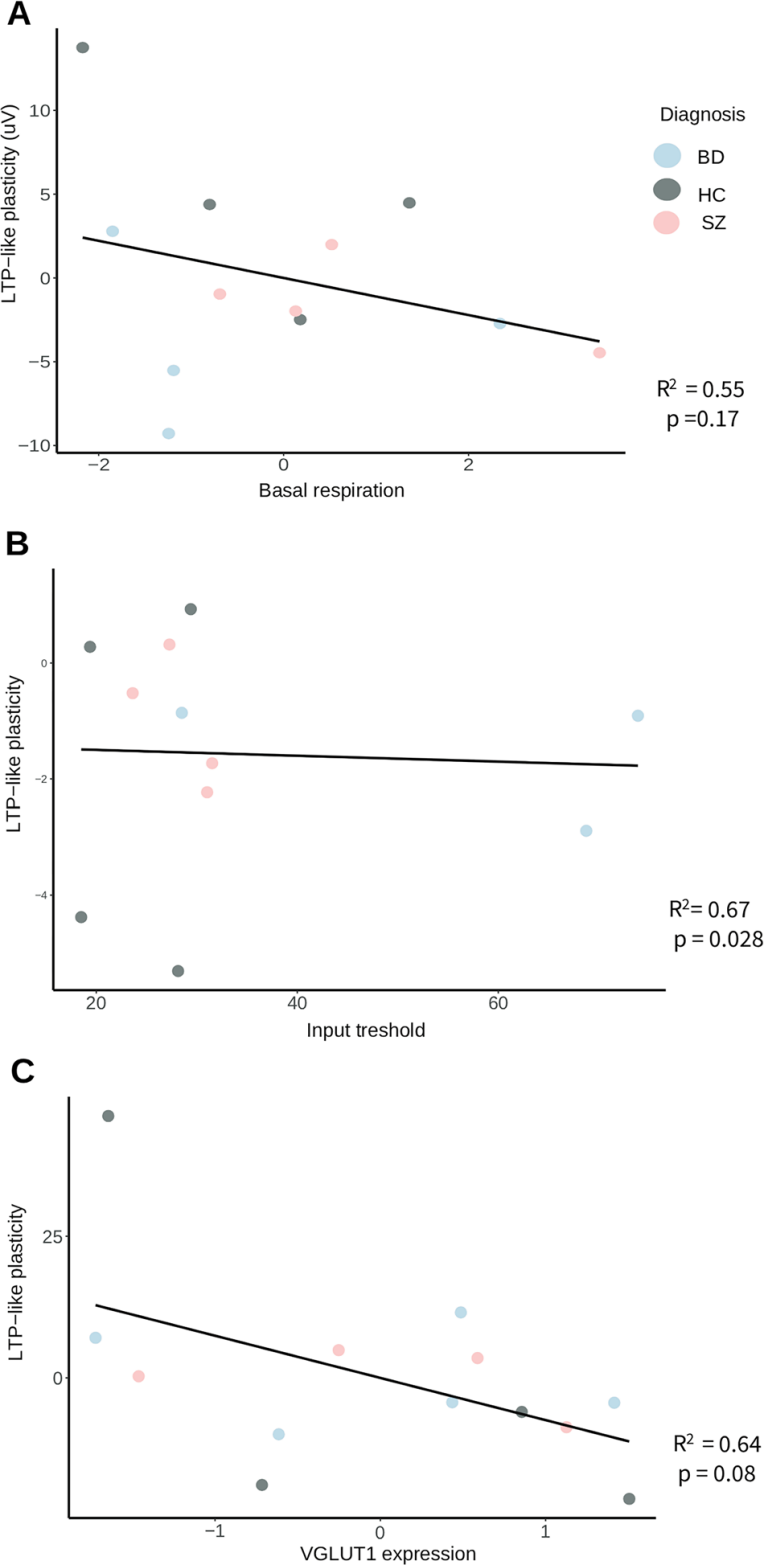
Associations between participants EEG and clinical variables with hCS read-outs. Partial regression plots of whole- sample associations between (**A)** LTP-like plasticity and hCS basal respiration. (**B**) LTP-like plasticity and hCS Input threshold. (**C**) LTP-like plasticity and hCS VGLUT1 expression. LTP long term potentiation-like plasticity, VGLUT1 vesicular glutamate transporter type 1.

Among the hCS variables differing between diagnoses, LTP-like plasticity showed a positive association with basal respiration (*P* = 0.028), which, however, did not survive correction, as well as a trend level association in the same direction (*P* = 0.08) with VGLUT1 expression, whereas no significant associations were found with input threshold (Fig. 5, Supplementary Table 5). Regarding the secondary analyses, some interesting associations at *P* < 0.05 level was noted including a nominally positive association between LTP-like plasticity and ATP-respiration and between MMN and SST expression. However none of these associations survived corrections for multiple testing (Supplementary Table 6).

## Discussion

We combined in vivo EEG- and in vitro hCS-based methods to provide new insights into neural impairments in SZ and BD. First, we found evidence for LTP-like plasticity impairment in the patients with SZ or BD of the present study, consistent with larger studies published previously [8, 11, 12]. Second, IHC showed decreased levels of VGLUT1 levels in both SZ and BD hCS. Third, Seahorse bioenergetics showed decreased mitochondrial respiration in BD hCS. Finally, there was a nominal positive association between LTP-like plasticity of the study participants and basal respiration measured on the hCS across the whole sample.

VGLUT1 is the most abundant glutamate transporter in the cerebral cortex and facilitates presynaptic exocytosis of glutamate into the synaptic cleft [52]. Since VGLUT1 expression level is the main factor determining quantal size in glutamatergic neurons [53], its reduced expression may impair synaptic function by reducing presynaptic released glutamate levels [54, 55]. Here we identified VGLUT1 in hCS as cytoplasmic vesicles, similarly to what is observed in rat neocortical neurons [56] Consistent with our findings, post-mortem studies have shown evidence of reduced VGLUT1 expression in the thalamus [57], anterior cingulate cortex [58], prefrontal cortex [58–60], and hippocampus [60] of subjects with SZ. Furthermore, VGLUT1 deficit has been reported in the medial temporal lobe in subjects with BD and major depression [61] and in dorsolateral prefrontal cortex from postmortem brains of BD individuals [59]. Despite some studies on postmortem brain tissue have failed in finding alterations in VGLUT1 levels in SZ, a novel method combining in situ hybridization of VGLUT1 mRNA and IHC-labelling of NeuN has been able to detect decrease in VGLUT1 mRNA levels and decreased soma size in neurons from the dorsolateral prefrontal cortex of SZ patients [62]. Thus, our results provide further support for altered presynaptic glutamatergic neurotransmission in SZ and BD. The current finding that VGLUT1 levels are reduced in both SZ and BD hCS is striking, indicating that this protein alteration may be a common pathophysiological event occurring very early during neural development in both disorders.

Alterations in bioenergetics have been reported in postmortem brain tissue and animal models of SZ and BD [30], and more recently in iPSC models of these disorders [37, 63]. Despite our reduced sample size, we could still detect reduced OXPHOS activity and a significant deficit in basal respiration in BD hCS. In contrast, SZ hCS presented normal OXPHOS parameters and glycolysis. The present results are different from recent work in which no OXPHOS or ECAR alterations were found in BD organoids while a reduction of both parameters were observed in SZ organoids [64]. Possible factors for these discrepancies include differences in the neural differentiation protocol, maturation stages of the organoids used in the experiments, and the lack of stratification of BD patients according to lithium responders and those who are lithium non-responders. In our previous study, hCS from lithium non-responder BD patients presented OXPHOS deficit whereas hCS derived from lithium responder patients presented normal OXPHOS activity [37]. This is in line with the current results since four out of five BD hCS were generated from BD lithium non-responders. Interestingly, several models of SZ have presented increased glycolysis, and accumulation of its end-product, lactate [65]. Thus, we speculate that BD and SZ may present alterations in brain energy metabolism profile already during fetal brain development. Given that we could not identify bioenergetic changes in SZ hCS, and that notable OXPHOS differences were observed in BD hCS, expanding the sample size and designing experiments to elucidate the mechanisms behind the observed bioenergetic alterations using our hCS models are warranted.

IPSC-derived hCS are self-assembled cell aggregates exhibiting high organization and neuronal connectivity [66]. Neurons in the hCS generate action potentials, display excitatory and inhibitory postsynaptic currents [37], and form a neuronal network, whose activity can be measured by calcium imaging and extracellular field potential recordings [67]. In the present work, electrophysiology read-outs from the hCS cells support their neuronal features due to passive and active properties typical for neurons.

Analysis of spontaneous postsynaptic currents sEPSCs recorded in hCS neurons revealed significantly smaller amplitudes of sEPSCs in the SZ group, that might be an early indicator for changes in glutamatergic signalling. Although hCS neurons exhibited only rare spontaneous activity at this time point (DIV180), suggesting the presence of immature glutamatergic synaptic transmission, it would be valuable to confirm this through additional analyses in larger studies, as well as by testing responses to electrical or pharmacological stimulation.

Finally, we report that LTP-plasticity as elicited by tetanic visual stimulation in a VEP paradigm across the whole sample was associated with increased basal respiration in hCS cultured from the same participants. This is in agreement with works suggesting that normal mitochondrial function is critical for electrophysiological activity [68]. For example, provoking mitochondrial defects in interneurons of mice leads to increased gamma and theta frequency oscillation power and behavioural alterations compatible to psychiatric disorders [69]. In addition, in vitro isolated mitochondria transfer (IMT) improves neuronal differentiation in iPSC derived from SZ patients, whereas in vivo IMT prevents mitochondrial dysfunction and abnormal behaviour in a neurodevelopmental mouse model of SZ [70]. Regarding the human brain, an association between mitochondrial function and redox status measured in peripheral blood with brain function (i.e. cortical beta-oscillations) and behavioural function in healthy adult subjects has been reported [71]. Still, more studies are needed for a better understing of the mechanisms behind the influence of mitochondrial function on EEG signals across mental disorders and controls.

Limitations of this work include a relatively small sample size, given the difficulty of finding individuals with both EEG data and iPSCs available; however, this is comparable to previously published hCS studies. The limited statistical power of our study represents a caveat, especially for in vitro parameters displaying subtle but non-statistically significant differences. This caveat may underpower our study, particularly in relation to in vitro parameters showing only small differences that do not reach statistical significance between cases and controls, such as the subtle increase in electrophysiological current threshold (It) in SZ hCS, and minor differences in basal glycolysis, with a tendency toward increased values in SZ hCS and decreased values in BD hCS. Despite the hCS protocol we used produces predominantly glutamatergic neurons, as neuronal classification based on electrophysiological firing profiles is not feasible at this stage of development, we cannot rule out the possibility that a small number of interneurons were also recorded among the predominantly glutamatergic population. However, as neuronal classification based on electrophysiological firing profiles was not feasible at this stage of development, we cannot rule out the possibility that a small number of interneurons were also recorded among the predominantly glutamatergic population. Finally, using the hCS model constrains pathophysiological findings to the fetal period of human brain development, which corresponds to the transcriptomic profile of our hCS model [35]. However, since SZ and BD are neurodevelopmental disorders [72, 73], comparing findings from the hCS model with postmortem brain tissue from patients may help elucidate the sequence of pathophysiological events underlying these conditions. The lack of vascularization of hCS could limit the access of nutrients and lead to cell death in the core of hCS, yet if it is the case, this would be in the same magnititude among the experimental groups since hCS from patients and from controls presented similar values of mitochondrial parameters, except for reduced basal respiration in the BD group. The NMDAR hypofunction hypothesis posits that reduced NMDAR function of PV and SST interneurons leads to aberrant neural oscillations in SZ [74, 75], and these proteins are downregulated in post-mortem prefrontal cortex in SZ [76]. Difficulties in finding specific antibodies against GRIN2A, SST and PV for IHC analyses hampered our investigation of the post-synaptic N-methyl-D-aspartate Receptor (NMDAR) [77], and GABAergic interneurons, which contribute to excitation/inhibition balance at the synaptic level [78]. Still, despite of many caveats, it is worth mentioning that one of the greatest advantages of using iPSC-based models over postmortem brain tissue include the possibility to assessing live human brain cells, and the absence of any influence from medication on the experimental results. Given the promising results observed despite the limited number of samples, expanding this study to include a larger cohort of control individuals and those with SZ and BD is warranted.To conclude, our study provides new insights into neural alterations in SZ and BD, and suggests potential neurodevelopmental origins of BD and SZ through a common alteration in VGLUT1 and distinct alterations in bioenergetics in hCS. While one should be cautious when directly interpreting correlations between in vitro data obtained from immature hCS-derived cells and in vivo data from mature neurons in adult human brain tissue, brain organoids nevertheless represent the best currently available model for mimicking human brain development. However, fundamental differences remain between adult synaptic circuitry and developmentally immature hCS, which limits the interpretation of the organoid-EEG associations observed in our study. In the future, larger studies using increasingly sophisticated organoids, better aligned with the adult human condition, may hold promise for unraveling the mechanistic underpinnings of neurodevelopmental disorders.

## Supplementary information

Supplemental Material

Supplementary Figure 1

Supplementary Figure 2

Supplementary Figure 3

## Data Availability

De-identified data supporting the findings of this study are available from the corresponding author upon reasonable request. Access is subjected to approval by the relevant ethics committee and data protection regulations.

## References

[1] Freedman R. Schizophrenia. N Engl J Med. 2003;349:1738–49. 14585943 10.1056/NEJMra035458

[2] Belmaker RH. Bipolar disorder. N Engl J Med. 2004;351:476–86. 15282355 10.1056/NEJMra035354

[3] Lopez AD, Murray CC. The global burden of disease, 1990-2020. Nat Med. 1998;4:1241–3. 9809543 10.1038/3218

[4] PGC. Genomic dissection of bipolar disorder and schizophrenia, including 28 subphenotypes. Cell. 2018;173:1705–15.e1716. 29906448 10.1016/j.cell.2018.05.046 PMC6432650

[5] Trubetskoy V, Pardiñas AF, Qi T, Panagiotaropoulou G, Awasthi S, Bigdeli TB, et al. Mapping genomic loci implicates genes and synaptic biology in schizophrenia. Nature. 2022;604:502–8. 35396580 10.1038/s41586-022-04434-5 PMC9392466

[6] Mullins N, Forstner AJ, O’Connell KS, Coombes B, Coleman JRI, Qiao Z, et al. Genome-wide association study of more than 40,000 bipolar disorder cases provides new insights into the underlying biology. Nat Genet. 2021. 10.1038/s41588-021-00857-4 PMC8192451 34002096

[7] Luck SJ *An Introduction to the Event-Related Potential Technique*. The MIT Press: Cambridge, MA, 2005.

[8] Cavus I, Reinhart RM, Roach BJ, Gueorguieva R, Teyler TJ, Clapp WC, et al. Impaired visual cortical plasticity in schizophrenia. Biol Psychiatry. 2012;71:512–20. 22364738 10.1016/j.biopsych.2012.01.013 PMC3292767

[9] Cooke SF, Bear MF. Stimulus-selective response plasticity in the visual cortex: an assay for the assessment of pathophysiology and treatment of cognitive impairment associated with psychiatric disorders. Biol Psychiatry. 2012;71:487–95. 22019003 10.1016/j.biopsych.2011.09.006

[10] Normann C, Schmitz D, Furmaier A, Doing C, Bach M. Long-term plasticity of visually evoked potentials in humans is altered in major depression. Biol Psychiatry. 2007;62:373–80. 17240361 10.1016/j.biopsych.2006.10.006

[11] Elvsåshagen T, Moberget T, Boen E, Boye B, Englin NO, Pedersen PO, et al. Evidence for impaired neocortical synaptic plasticity in bipolar II disorder. Biol Psychiatry. 2012;71:68–74. 22036034 10.1016/j.biopsych.2011.09.026

[12] Valstad M, Roelfs D, Slapo NB, Timpe CMF, Rai A, Matziorinis AM, et al. Evidence for reduced long-term potentiation-like visual cortical plasticity in schizophrenia and bipolar disorder. Schizophr Bull. 2021;47:1751–60. 33963856 10.1093/schbul/sbab049 PMC8530383

[13] Naatanen R, Alho K. Mismatch negativity-a unique measure of sensory processing in audition. Int J Neurosci. 1995;80:317–37. 7775056 10.3109/00207459508986107

[14] Thiebes S, Leicht G, Curic S, Steinmann S, Polomac N, Andreou C, et al. Glutamatergic deficit and schizophrenia-like negative symptoms: new evidence from ketamine-induced mismatch negativity alterations in healthy male humans. J Psychiatry Neurosci. 2017;42:273–83. 28556775 10.1503/jpn.160187 PMC5487274

[15] Javitt DC, Steinschneider M, Schroeder CE, Arezzo JC. Role of cortical N-methyl-D-aspartate receptors in auditory sensory memory and mismatch negativity generation: implications for schizophrenia. Proc Natl Acad Sci USA. 1996;93:11962–7. 8876245 10.1073/pnas.93.21.11962 PMC38166

[16] de Sousa RT, Machado-Vieira R, Zarate CA Jr, Manji HK. Targeting mitochondrially mediated plasticity to develop improved therapeutics for bipolar disorder. Expert Opin Ther Targets. 2014;18:1131–47. 25056514 10.1517/14728222.2014.940893 PMC4180305

[17] Hermens DF, Chitty KM, Kaur M. Mismatch negativity in bipolar disorder: A neurophysiological biomarker of intermediate effect?. Schizophr Res. 2018;191:132–9. 28450056 10.1016/j.schres.2017.04.026

[18] Erickson MA, Ruffle A, Gold JM. A meta-analysis of mismatch negativity in schizophrenia: from clinical risk to disease specificity and progression. Biol Psychiatry. 2016;79:980–7. 26444073 10.1016/j.biopsych.2015.08.025 PMC4775447

[19] Light GA, Swerdlow NR, Thomas ML, Calkins ME, Green MF, Greenwood TA, et al. Validation of mismatch negativity and P3a for use in multi-site studies of schizophrenia: characterization of demographic, clinical, cognitive, and functional correlates in COGS-2. Schizophr Res. 2015;163:63–72. 25449710 10.1016/j.schres.2014.09.042 PMC4382452

[20] Rangaraju V, Calloway N, Ryan TA. Activity-driven local ATP synthesis is required for synaptic function. Cell. 2014;156:825–35. 24529383 10.1016/j.cell.2013.12.042 PMC3955179

[21] Logothetis NK, Pauls J, Augath M, Trinath T, Oeltermann A. Neurophysiological investigation of the basis of the fMRI signal. Nature. 2001;412:150–7. 11449264 10.1038/35084005

[22] Magistretti PJ, Allaman I. A cellular perspective on brain energy metabolism and functional imaging. Neuron. 2015;86:883–901. 25996133 10.1016/j.neuron.2015.03.035

[23] Pellerin L, Magistretti PJ. Sweet sixteen for ANLS. Journal of cerebral blood flow and metabolism : official journal of the International Society of Cerebral Blood Flow and Metabolism. 2012;32:1152–66. 22027938 10.1038/jcbfm.2011.149 PMC3390819

[24] Du F, Cooper AJ, Thida T, Sehovic S, Lukas SE, Cohen BM, et al. In vivo evidence for cerebral bioenergetic abnormalities in schizophrenia measured using 31P magnetization transfer spectroscopy. JAMA Psychiatry. 2014;71:19–27. 24196348 10.1001/jamapsychiatry.2013.2287 PMC7461723

[25] Du F, Yuksel C, Chouinard VA, Huynh P, Ryan K, Cohen BM, et al. Abnormalities in high-energy phosphate metabolism in first-episode bipolar disorder measured using (31)P-magnetic resonance spectroscopy. Biol Psychiatry. 2018;84:797–802. 28527566 10.1016/j.biopsych.2017.03.025 PMC5632123

[26] Sarnyai Z, Ben-Shachar D. Schizophrenia, a disease of impaired dynamic metabolic flexibility: A new mechanistic framework. Psychiatry Res. 2024;342:116220. 39369460 10.1016/j.psychres.2024.116220

[27] McCullumsmith RE, Clinton SM, Meador-Woodruff JH. Schizophrenia as a disorder of neuroplasticity. Int Rev Neurobiol. 2004;59:19–45. pp. 15006483 10.1016/S0074-7742(04)59002-5

[28] Hjelm BE, Rollins B, Mamdani F, Lauterborn JC, Kirov G, Lynch G, et al. Evidence of mitochondrial dysfunction within the complex genetic etiology of schizophrenia. Mol Neuropsychiatry. 2015;1:201–19. 26550561 10.1159/000441252 PMC4635522

[29] Breen MS, Dobbyn A, Li Q, Roussos P, Hoffman GE, Stahl E, et al. Global landscape and genetic regulation of RNA editing in cortical samples from individuals with schizophrenia. Nat Neurosci. 2019;22:1402–12. 31455887 10.1038/s41593-019-0463-7 PMC6791127

[30] Manji H, Kato T, Di Prospero NA, Ness S, Beal MF, Krams M, et al. Impaired mitochondrial function in psychiatric disorders. Nat Rev Neurosci. 2012;13:293–307. 22510887 10.1038/nrn3229

[31] Lancaster MA, Knoblich JA. Organogenesis in a dish: modeling development and disease using organoid technologies. Science (New York, NY). 2014;345:1247125. 10.1126/science.1247125 25035496

[32] Lancaster MA, Renner M, Martin CA, Wenzel D, Bicknell LS, Hurles ME, et al. Cerebral organoids model human brain development and microcephaly. Nature. 2013;501:373–9. 23995685 10.1038/nature12517 PMC3817409

[33] Paşca AM, Sloan SA, Clarke LE, Tian Y, Makinson CD, Huber N, et al. Functional cortical neurons and astrocytes from human pluripotent stem cells in 3D culture. Nat Methods. 2015;12:671–8. 26005811 10.1038/nmeth.3415 PMC4489980

[34] Quadrato G, Nguyen T, Macosko EZ, Sherwood JL, Min Yang S, Berger DR, et al. Cell diversity and network dynamics in photosensitive human brain organoids. Nature. 2017;545:48–53. 28445462 10.1038/nature22047 PMC5659341

[35] Akkouh IA, Ueland T, Szabo A, Hughes T, Smeland OB, Andreassen OA. et al. Longitudinal transcriptomic analysis of human cortical spheroids identifies axonal dysregulation in the prenatal brain as a mediator of genetic risk for schizophrenia. Biol Psychiatry. 2024;95:687–98. 37661009 10.1016/j.biopsych.2023.08.017

[36] Yang G, Ullah HMA, Parker E, Gorsi B, Libowitz M, Maguire C. et al. Neurite outgrowth deficits caused by rare PLXNB1 mutation in pediatric bipolar disorder. Mol Psychiatry. 2023;28:2525–39. 37032361 10.1038/s41380-023-02035-w

[37] Osete JR, Akkouh IA, Ievglevskyi O, Vandenberghe M, de Assis DR, Ueland T, et al. Transcriptional and functional effects of lithium in bipolar disorder iPSC-derived cortical spheroids. Mol Psychiatry. 2023;28:3033–43. 36653674 10.1038/s41380-023-01944-0 PMC10615757

[38] Notaras M, Lodhi A, Dündar F, Collier P, Sayles NM, Tilgner H, et al. Schizophrenia is defined by cell-specific neuropathology and multiple neurodevelopmental mechanisms in patient-derived cerebral organoids. Mol Psychiatry. 2022;27:1416–34. 34789849 10.1038/s41380-021-01316-6 PMC9095467

[39] Kathuria A, Lopez-Lengowski K, Jagtap SS, McPhie D, Perlis RH, Cohen BM, et al. Transcriptomic landscape and functional characterization of induced pluripotent stem cell-derived cerebral organoids in schizophrenia. JAMA Psychiatry. 2020;77:745–54. 32186681 10.1001/jamapsychiatry.2020.0196 PMC7081156

[40] Stachowiak EK, Benson CA, Narla ST, Dimitri A, Chuye LEB, Dhiman S, et al. Cerebral organoids reveal early cortical maldevelopment in schizophrenia-computational anatomy and genomics, role of FGFR1. Transl Psychiatry. 2017;7:6. 30446636 10.1038/s41398-017-0054-x PMC5802550

[41] Kathuria A, Lopez-Lengowski K, Vater M, McPhie D, Cohen BM, Karmacharya R. Transcriptome analysis and functional characterization of cerebral organoids in bipolar disorder. Genome Med. 2020;12:34. 32306996 10.1186/s13073-020-00733-6 PMC7168850

[42] Simonsen C, Sundet K, Vaskinn A, Birkenaes AB, Engh JA, Faerden A, et al. Neurocognitive dysfunction in bipolar and schizophrenia spectrum disorders depends on history of psychosis rather than diagnostic group. Schizophr Bull. 2011;37:73–83. 19443616 10.1093/schbul/sbp034 PMC3004191

[43] Pentz AB, O’Connel KS, van Jole O, Timpe CMF, Slapo NB, Melle I, et al. Mismatch negativity and polygenic risk scores for schizophrenia and bipolar disorder. Schizophr Res. 2024;264:314–26. 38215567 10.1016/j.schres.2024.01.013

[44] Delorme A, Makeig S. EEGLAB: an open-source toolbox for analysis of single trial EEG dynamics. J Neurosci Methods. 2004;134:139–121. 10.1016/j.jneumeth.2003.10.009 15102499

[45] Bigdely-Shamlo N, Mullen T, Kothe C, Su KM, Robbins KA. The PREP pipeline: standardized preprocessing for large-scale EEG analysis. Front Neuroinform. 2015;9:16. 26150785 10.3389/fninf.2015.00016 PMC4471356

[46] Pion-Tonachini L, Kreutz-Delgado K, Makeig S. ICLabel: An automated electroencephalographic independent component classifier, dataset, and website. Neuroimage. 2019;198:181–97. 31103785 10.1016/j.neuroimage.2019.05.026 PMC6592775

[47] Valstad M, Moberget T, Roelfs D, Slapo NB, Timpe CMF, Beck D, et al. Experience-dependent modulation of the visual evoked potential: Testing effect sizes, retention over time, and associations with age in 415 healthy individuals. Neuroimage. 2020;223:117302. 32828930 10.1016/j.neuroimage.2020.117302

[48] Garrido MI, Friston KJ, Kiebel SJ, Stephan KE, Baldeweg T, Kilner JM. The functional anatomy of the MMN: a DCM study of the roving paradigm. Neuroimage. 2008;42:936–44. 18602841 10.1016/j.neuroimage.2008.05.018 PMC2640481

[49] Baldeweg T, Klugman A, Gruzelier J, Hirsch SR. Mismatch negativity potentials and cognitive impairment in schizophrenia. Schizophr Res. 2004;69:203–17. 15469194 10.1016/j.schres.2003.09.009

[50] Yoon SJ, Elahi LS, Pașca AM, Marton RM, Gordon A, Revah O, et al. Reliability of human cortical organoid generation. Nat Methods. 2019;16:75–8. 30573846 10.1038/s41592-018-0255-0 PMC6677388

[51] Pentz AB, Timpe CMF, Normann EM, Slapo NB, Melle I, Lagerberg TV, et al. Mismatch negativity in schizophrenia spectrum and bipolar disorders: Group and sex differences and associations with symptom severity. Schizophr Res. 2023;261:80–93. 37716205 10.1016/j.schres.2023.09.012

[52] Moriyama Y, Yamamoto A. Glutamatergic chemical transmission: look! Here, there, and anywhere. J Biochem. 2004;135:155–63. 15047716 10.1093/jb/mvh018

[53] Wilson NR, Kang J, Hueske EV, Leung T, Varoqui H, Murnick JG, et al. Presynaptic regulation of quantal size by the vesicular glutamate transporter VGLUT1. J Neurosci. 2005;25:6221–34. 15987952 10.1523/JNEUROSCI.3003-04.2005 PMC6725055

[54] Liguz-Lecznar M, Skangiel-Kramska J. Vesicular glutamate transporters (VGLUTs): the three musketeers of glutamatergic system. Acta Neurobiol Exp (Wars). 2007;67:207–18. 17957901 10.55782/ane-2007-1649

[55] Balschun D, Moechars D, Callaerts-Vegh Z, Vermaercke B, Van Acker N, Andries L, et al. Vesicular glutamate transporter VGLUT1 has a role in hippocampal long-term potentiation and spatial reversal learning. Cereb Cortex. 2010;20:684–93. 19574394 10.1093/cercor/bhp133

[56] Berry CT, Sceniak MP, Zhou L, Sabo SL. Developmental up-regulation of vesicular glutamate transporter-1 promotes neocortical presynaptic terminal development. PLoS ONE. 2012;7:e50911. 23226425 10.1371/journal.pone.0050911 PMC3511412

[57] Smith RE, Haroutunian V, Davis KL, Meador-Woodruff JH. Vesicular glutamate transporter transcript expression in the thalamus in schizophrenia. Neuroreport. 2001;12:2885–7. 11588596 10.1097/00001756-200109170-00026

[58] Oni-Orisan A, Kristiansen LV, Haroutunian V, Meador-Woodruff JH, McCullumsmith RE. Altered Vesicular Glutamate Transporter Expression in the Anterior Cingulate Cortex in Schizophrenia. Biol Psychiatry. 2008;63:766–75. 18155679 10.1016/j.biopsych.2007.10.020 PMC2669959

[59] Gilabert-Juan J, Varea E, Guirado R, Blasco-Ibáñez JM, Crespo C, Nácher J. Alterations in the expression of PSA-NCAM and synaptic proteins in the dorsolateral prefrontal cortex of psychiatric disorder patients. Neurosci Lett. 2012;530:97–102. 23022470 10.1016/j.neulet.2012.09.032

[60] Eastwood SL, Harrison PJ. Decreased expression of vesicular glutamate transporter 1 and complexin II mRNAs in schizophrenia: further evidence for a synaptic pathology affecting glutamate neurons. Schizophr Res. 2005;73:159–72. 15653259 10.1016/j.schres.2004.05.010

[61] Uezato A, Meador-Woodruff JH, McCullumsmith RE. Vesicular glutamate transporter mRNA expression in the medial temporal lobe in major depressive disorder, bipolar disorder, and schizophrenia. Bipolar Disord. 2009;11:711–25. 19839996 10.1111/j.1399-5618.2009.00752.x

[62] Schoonover KE, Miller NE, Fish KN, Lewis DA. Scaling of smaller pyramidal neuron size and lower energy production in schizophrenia. Neurobiol Dis. 2024;191:106394. 38176569 10.1016/j.nbd.2023.106394 PMC10898364

[63] Ni P, Noh H, Park GH, Shao Z, Guan Y, Park JM, et al. iPSC-derived homogeneous populations of developing schizophrenia cortical interneurons have compromised mitochondrial function. Mol Psychiatry. 2020;25:2873–88. 31019265 10.1038/s41380-019-0423-3 PMC6813882

[64] Kathuria A, Lopez-Lengowski K, McPhie D, Cohen BM, Karmacharya R. Disease-specific differences in gene expression, mitochondrial function and mitochondria-endoplasmic reticulum interactions in iPSC-derived cerebral organoids and cortical neurons in schizophrenia and bipolar disorder. Discover mental health. 2023;3:8. 36915374 10.1007/s44192-023-00031-8 PMC9998323

[65] Sullivan CR, Mielnik CA, Funk A, O’Donovan SM, Bentea E, Pletnikov M, et al. Measurement of lactate levels in postmortem brain, iPSCs, and animal models of schizophrenia. Sci Rep. 2019;9:5087. 30911039 10.1038/s41598-019-41572-9 PMC6433855

[66] Sakaguchi H, Ozaki Y, Ashida T, Matsubara T, Oishi N, Kihara S, et al. Self-organized synchronous calcium transients in a cultured human neural network derived from cerebral organoids. Stem Cell Reports. 2019;13:458–73. 31257131 10.1016/j.stemcr.2019.05.029 PMC6739638

[67] Trujillo CA, Gao R, Negraes PD, Gu J, Buchanan J, Preissl S, et al. Complex oscillatory waves emerging from cortical organoids model early human brain network development. Cell Stem Cell. 2019;25:558–69.e557. 31474560 10.1016/j.stem.2019.08.002 PMC6778040

[68] Pekkurnaz G, Wang X. Mitochondrial heterogeneity and homeostasis through the lens of a neuron. Nature Metabolism. 2022;4:802–12. 35817853 10.1038/s42255-022-00594-w PMC11151822

[69] Inan M, Zhao M, Manuszak M, Karakaya C, Rajadhyaksha AM, Pickel VM, et al. Energy deficit in parvalbumin neurons leads to circuit dysfunction, impaired sensory gating and social disability. Neurobiol Dis. 2016;93:35–46. 27105708 10.1016/j.nbd.2016.04.004

[70] Robicsek O, Ene HM, Karry R, Ytzhaki O, Asor E, McPhie D, et al. Isolated mitochondria transfer improves neuronal differentiation of schizophrenia-derived induced pluripotent stem cells and rescues deficits in a rat model of the disorder. Schizophr Bull. 2018;44:432–42. 28586483 10.1093/schbul/sbx077 PMC5814822

[71] Spooner RK, Taylor BK, Ahmad IM, Dyball KN, Emanuel K, Fox HS, et al. Neural oscillatory activity serving sensorimotor control is predicted by superoxide-sensitive mitochondrial redox environments. Proc Natl Acad Sci USA. 2021;118:e2104569118. 34686594 10.1073/pnas.2104569118 PMC8639326

[72] Birnbaum R, Weinberger DR. Genetic insights into the neurodevelopmental origins of schizophrenia. Nat Rev Neurosci. 2017;18:727–40. 29070826 10.1038/nrn.2017.125

[73] Kloiber S, Rosenblat JD, Husain MI, Ortiz A, Berk M, Quevedo J, et al. Neurodevelopmental pathways in bipolar disorder. Neurosci Biobehav Rev. 2020;112:213–26. 32035092 10.1016/j.neubiorev.2020.02.005

[74] Chung DW, Geramita MA, Lewis DA. Synaptic variability and cortical gamma oscillation power in schizophrenia. Am J Psychiatry. 2022;179:277–87. 35360919 10.1176/appi.ajp.2021.21080798 PMC9580070

[75] Van Derveer AB, Bastos G, Ferrell AD, Gallimore CG, Greene ML, Holmes JT, et al. A role for somatostatin-positive interneurons in neuro-oscillatory and information processing deficits in schizophrenia. Schizophr Bull. 2021;47:1385–98. 33370434 10.1093/schbul/sbaa184 PMC8379548

[76] Dienel SJ, Fish KN, Lewis DA The nature of prefrontal cortical GABA neuron alterations in schizophrenia: markedly lower somatostatin and parvalbumin gene expression without missing neurons. *The American journal of psychiatry* 2023**:** 180:495-507. 10.1176/appi.ajp.20220676 PMC10330559 37073488

[77] Javitt DC, Kantrowitz JT. The glutamate/N-methyl-d-aspartate receptor (NMDAR) model of schizophrenia at 35: On the path from syndrome to disease. Schizophr Res. 2022;242:56–61. 35125283 10.1016/j.schres.2022.01.013

[78] Booker SA, Wyllie DJA. NMDA receptor function in inhibitory neurons. Neuropharmacology. 2021;196:108609. 34000273 10.1016/j.neuropharm.2021.108609

