# Supplementary material for: Investigating neural impairments in psychotic disorders using electroencephalography and cortical spheroids
Source: Transl Psychiatry. 2026 Feb 17;16:114. doi: 10.1038/s41398-026-03863-4 (PMC12949052; doi:10.1038/s41398-026-03863-4)
Supplement: Supplementary file 1 — Supplemental Material [file 41398_2026_3863_MOESM1_ESM.docx]

Supplementary Material for

**Investigating neural impairments in psychotic disorders using electroencephalography and cortical spheroids**

Denis Reis de Assis^1,2^, PhD; Atle Bråthen Pentz^1^, PhD; Jordi Requena Osete^1,2^, PhD; Oleksandr Ievglevskyi^1^, PhD candidate; Matthieu Vandenberghe^1,2^, PhD; Ibrahim Ahmed Akkouh^1,2^, PhD; Tuomo Mäki-Marttunen^3,4^, PhD; Erik G. Jönsson^1,5^, PhD; Ole A. Andreassen^1^, PhD; Srdjan Djurovic^1,2^, PhD; Elena Kondratskaya^1,2*^, PhD; Torbjørn Elvsåshagen^1,6,7*^, PhD

^1^ Center for Precision Psychiatry Division of Mental Health and Addiction, Oslo University Hospital & Institute of Clinical Medicine, University of Oslo, and Division of Mental Health and Addiction, Oslo University Hospital, Oslo, Norway

^2^ Department of Medical Genetics, Oslo University Hospital, Oslo, Norway

^3^ Faculty of Medicine and Health Technology, Tampere University, Tampere, Finland

^4^ Department of Biosciences, University of Oslo, Oslo, Norway

^5^Centre for Psychiatric Research, Department of Clinical Neuroscience, Karolinska Institutet & Stockholm Health Care Sciences, Stockholm Region, Stockholm, Sweden

^6^ Department of Neurology, Oslo University Hospital, Oslo, Norway

^7^ Department of Behavioural Medicine, Institute of Basic Medical Sciences, University of Oslo, Oslo, Norway

* Shared senior authorship

**Author correspondence:** Denis Reis de Assis, Ph.D., Department of Medical Genetics, Oslo University Hospital, Oslo, Norway; Centre for Precision Psychiatry, Institute of Clinical Medicine, University of Oslo, Norway. Electronic address: denisre@medisin.uio.no

**Methods**

**Clinical assessments**

The patients were assessed by a trained clinician with interviews for the Diagnostic and Statistical Manual of Mental Disorders, Fourth Edition (DSM-IV; SCID-I)^1^ and met DSM-IV criteria for a SZ spectrum disorder or a BD spectrum disorder. Symptoms were assessed with the Positive And Negative Syndrome Scale (PANSS)^2^, the Montgomery-Åsberg Depression Rating Scale (MADRS)^3^, and the Young Mania Rating Scale (YMRS)^4^. The Intelligence Quotient (IQ) were assessed using Wechsler Abbreviated Scale of Intelligence (WASI)^5^. Neurological illness, autism spectrum disorder, brain injury or history of severe head trauma with loss of consciousness, other significant medical illness affecting brain function, and IQ < 70 were exclusion criteria for patients and controls. The CTRL also underwent the Primary Care Evaluation of Mental Disorders^6^ and these were additional exclusion criteria for the controls: a) a history of SZ, BD, or major depressive disorder (MDD) and b) having a first degree relative with SZ, BD, or MDD.

**EEG acquisition and preprocessing**

Scalp EEG data was amplified (-3dB at 417Hz low-pass, DC-coupled) and digitized (2048Hz) from 72 Ag/AgCl active electrodes, including 64 active channels positioned according to the international 10-5 system, using a Biosemi Active-Two amplifier (BioSemi, Amsterdam, The Netherlands). All electrode offsets were kept below +/-30µV. The offline analyses were conducted in Matlab 2017a (Mathworks Inc., Natick, MA) using EEGLAB and in-house scripts. The data was first low-pass filtered at 40Hz, then downsampled to 512Hz, subjected to a high-pass filter at 0.5Hz, and re-referenced to the average of left and right mastoids. Line noise and bad channels were removed using the PREP pipeline^7^ and affected channels were interpolated using a robust average reference. The mean number of interpolated channels was 3.6 (standard deviation (SD) 1.6, range 1-6). Epochs were then extracted from -100ms to 400ms relative to stimulus onset. Independent component analysis (ICA) was conducted using binica and independent components representing artefacts were identified using ICLabel. Components with label probabilities < 30% brain and > 50% non-brain were removed, thus resulting in the removal of 25 components on average (SD 4.3, range 14-33). Epochs were then baseline-corrected by subtracting the mean amplitude from -50ms to 0ms relative to stimulus onset and epochs with amplitudes exceeding ±100 mA were rejected.

**Mismatch negativity paradigm**

The participants were seated in a comfortable chair during the EEG data collection. Hearing threshold was tested binaurally using a pure sinusoidal 1000Hz tone and participants with a threshold of >40dB were excluded. MMN was obtained by a roving paradigm, scripted and presented using PsychToolbox in Matlab while the participants were reading a magazine to divert attention from the presented stimuli. Pure sinusoidal tones were presented binaurally (80dB, 5ms rise and fall times) by earphones (ER-2, Etymotic Research, Inc., Elk Grove Village, IL, USA) with a fixed stimulus onset asynchrony of 400ms. In the roving paradigm, trains of identical standard auditory stimuli with regards to pitch and duration were presented in a pseudorandom fashion with 2, 6, or 18 repetitions alternating with a new train of stimuli with different physical properties, thus resulting in the first tone of a new train acting as a deviant stimulus relative to last tone of the previous stimulus train. The pitch of the employed 24 tones ranged from 700-1250Hz, and the duration of the tones was either 50 or 100ms. For each new tone sequence, both the frequency and the duration of the tones changed, thus making the first tone of a new sequence a “double deviant”, accounting for 11.5% of the total number of stimuli. ERP waves from the FCZ electrode were calculated for all deviants and the immediately preceding standard stimulus by extracting the mean amplitudes in the 100 to 200ms post-stimulus interval and MMN was calculated by subtracting the averaged standard stimulus ERP from that of the deviant. The mean number of trials for the grand average MMN was 239.1 (SD 13.0, range 200-261)

**Cortical spheroids generation**

Induced pluripotent stem cells (iPSCs) were transferred to a 6-well Aggrewell 800 (18×10^6^ cells/well), centrifuged (3 min /100g), and incubated at 37 °C with 5% CO_2_ for 24 hours in Essential 8 Flex medium supplemented with 10µM Y-27632. Spheroids were cultured in ultra-low-attachment plastic dishes in Essential 6 medium supplemented with 10 μM SB-431542, 2.5 μM dorsomorphin, and 2.5 μM XAV-93 until day 6, and then changed to a neural medium supplemented with 20 ng/ml EGF and bFGF for 19 days until day 24. From day 24 until day 43 the neural medium was supplemented with 20 ng/ml BDNF and NT3 to promote neural differentiation. Human cortical spheroids (hCS) were generated from the iPSCs following the method of Yoon and colleagues^8^. After dissociating iPSCs with accutase (Sigma, A6964) in Essential 8 Flex medium supplemented with 10µM Y-27632 (Miltenyi Biotec), 18×10^6^ cells/well were added to a 6-well Aggrewell 800 (STEMCELL Technologies, 34815), centrifuged (3 min at 100g), and incubated at 37 °C with 5% CO_2_ for 24h. Spheroids were collected and cultured in ultra-low-attachment plastic dishes (Thermo Fisher, 15297905) in Essential 6 medium (Life Technologies, A1516401) supplemented with SMAD inhibitors (SB-431542 10 μM, Tocris, 1614) and dorsomorphin 2.5 μM, Sigma-Aldrich, P5499) and the Wnt inhibitor XAV-939 (2.5 μM, Tocris, 3748). The medium was changed daily until day 6, when the medium was shifted to a neural medium: Neurobasal A (Life Technologies, 10888), GlutaMax (1:100, Life Technologies, 35050) and B-27 supplement without vitamin A (Life Technologies, 12587). The neural medium was supplemented for 19 days until day 24 with 20 ng/ml EGF (R&D Systems, 236-EG) and 20 ng/ml bFGF (R&D Systems, 233-FB), with medium changed daily in the first 10 days and every other day for the following 9 days. From day 24 until day 43 the neural medium was supplemented with 20 ng/ml BDNF (Peprotech, 450-02) and 20 ng/ml NT3 (Peprotech, 450-03) to promote neural differentiation and the medium was changed every other day. From day 43 onwards, only the neural medium without growth factors was changed every 3-4 days.

**RNA sequencing and cell type deconvolution**

Total RNA was extracted from hCS using the RNeasy Plus Mini Kit (Qiagen). Library preparation and paired-end RNA-sequencing were carried out at the Norwegian High-Throughput Sequencing Centre (www.sequencing.uio.no). Briefly, libraries were prepared with the TruSeq Stranded mRNA kit from Illumina. The prepared samples were then sequenced in two batches on Illumina NovaSeq S4/X at an average depth of ~62 million reads per sample, using a read length of 150 bp and an insert size of ~350 bp.

Raw sequencing reads were quality assessed with FastQC (Babraham Institute) and further processed with Trimmomatic V0.32^9^. Hisat2^10^ was then used to map the trimmed reads to the human GRCh38 reference genome. To quantify gene expression levels, mapped reads were summarized at the gene level using featureCounts^11^ guided by Ensembl annotations.

Computational estimation of cell type fractions (deconvolution) was performed with InstaPrism^12^, a reimplementation of a Bayesian approach to the estimation of cell type composition^13^. A recent primary tissue single-cell dataset of human brain development was used to construct cell type signatures^14^. In accordance with the spatiotemporal identity of our hCS ^15^, only cells from frontocortical regions of the second and third trimesters were used for signature construction.

**Cryosectioning and immunohistochemistry**

One hCS per participant was fixed in 4% paraformaldehyde in PBS O/N at 4°C, transferred to 30% sucrose for 24h, embedded into optimum cutting temperature (OCT) compound (Sakura Finetek, #4583) and stored at -80°C. The hCS were then cut in sections 10 µm thick with a cryostat (Leica) for immunohistochemistry analysis. Sections were washed with PBS and blocked in PBS with 6% donkey serum, 0.2% Triton X-100 diluted in PBS for 1 hour at room temperature. Sections were incubated with primary antibodies in incubation buffer: PBS with 6% donkey serum, 0,04% TritonX100 O/N at 4°C in a humidified chamber. After washing with PBS, sections were incubated with secondary antibodies in incubation buffer for 2 hours at 37°C in a humidified chamber. Primary and secondary antibodies and dilutions used are specified in the Supplementary Table 1. Nuclei were stained with 4’,6-diamidino-2-phenylindole (DAPI, Roche, 10236276001) and then cover glasses (VWR, ECN631-1575) were mounted on top with Fluoromount-G (Invitrogen, #00-4958-02). Images were acquired using a Zeiss LSM 700 confocal microscope and processed using Fiji software. Fields based on uniform DAPI staining were selected. 300 cells were analysed for each marker from all lines. VGLUT1 presented as cytoplasmic vesicles, which were counted. The results were expressed as % of positive cells, being calculated based on the number of VGLUT1 vesicles over the total number of cells (based on DAPI staining). MAP2 was measured by fluorescence intensity using the Fiji software. We also tested antibodies against the common markers for GABAergic interneurons GRIN2A, SST and PV, however the staining resulted in unspecific signals, and were not included in this work.

***In Vitro* electrophysiology**

To access multiple neuronal characteristics in hCS neurons, whole-cell patch-clamp was applied in one hCS per cell donor. The hCS were cut into 150-200 µm slices by vibrating microtome (VT1000S; Leica) in the ASCF containing (in mM): 150 NaCl, 5 KCl, 11 glucose, 1 MgSO4, 2 CaCl2, 10 HEPES; pH 7.4; 310-320 mOsm/L. Slices were allowed to recover for 30 min at room temperature in ASCF and transferred to submersion recording chamber perfused with standard ASFC for recording.

The cortical neurons in hCS slices were visually identified with an up-right infrared-differential interference contrast (IR-DIC) microscope (Olympus BX51WI) and captured with a CoolSNAP EZ with SSD ICX285 (Photometrics) video camera. Fluorescent dye Alexa Fluor 488 was added to the pipette solution during patch-clamp experiments at a concentration of 10 μM in some experiemnts. Fluorescence imaging was performed under TIRF microscopy (Olympus BX51WI). Fluorescence excitation was at 482(±20) nm using a 40X water-immersion objective (numerical aperture 0.8, Olympus), and a xenon light source (Sutter Instruments) with shutter control (VCM-D1, Uniblitz, Vincent Associates). The images were acquired with a 0.9 s exposure. Image analysis was conducted using Fiji/ImageJ Software (http://fiji.sc, and GIMP (https://www.gnu.org).

Patch electrodes were fabricated from borosilicate glass capillaries with an outer diameter of 1.5 mm (Sutter Instruments) using an automated puller (PC-10; Narishige). The internal pipette solution contained (in mM): 130 K-methylsulfate, 5 NaCl, 1 CaCl2, 20 HEPES, 0.02 EGTA, 3 Mg-ATP, 0.4 Na3GTP-GTP. Pipette solutions were freshly made and filtered before the experiments (20 µm pore), osmolarity was 290 mOsm/L, and pipette resistance was 8-12 MOhm.

All recordings were performed using a Multi-Clamp 700B amplifier (Molecular Devices). Recordings and pre-processing of data were made with WinWCP (University of Strathclyde). The signals were typically low-pass filtered with a corner frequency (_x0005_3 dB) of 3 kHz and sampled at 6 kHz by DigiData 1322A (Molecular Devices). Data analysis was performed with p-CLAMP 10 (Molecular Devices) and OriginLab 8 (OriginLab Corp.).

Recordings were performed in a voltage-clamp (recording of Na^+^/K^+^ currents or current-clamp mode for action potential activation (AP) due to step current injection applying the standard protocols, step increment 5 pA unless other is indicated. Spontaneous excitatory postsynaptic currents (sEPSC) were recorded in voltage-clamp mode Vh=-70mV as was previously described ^16^. Due to estimated chloride reverse potential calculated with Nernst equation we excluded the potential impact iPSCs to postsynaptic currents recording at mentioned holding potential. sEPSCs were not separated as action potential driven and miniature synaptic currents.

For voltage clamp data analyses, recordings from 3-6 neurons per line were used for the CTRL vs. SZ comparison, and 2-3 neurons per line for the CTRL vs. BD comparison. For current clamp data and spontaneous excitatory postsynaptic current analyses data were combined for each group. A total of 89 neurons were recorded for all experimental groups.

**References**

1. Association AP. *Diagnostic and Statistical Manual of Mental Disorders*. 4th edn, 2000, 943pp.

2. Kay SR, Fiszbein A, Opler LA. The positive and negative syndrome scale (PANSS) for schizophrenia. *Schizophrenia bulletin* 1987; **13**(2)**:** 261-276.

3. Montgomery SA, Asberg M. A new depression scale designed to be sensitive to change. *Br J Psychiatry* 1979; **134:** 382-389.

4. Young RC, Biggs JT, Ziegler VE, Meyer DA. A rating scale for mania: reliability, validity and sensitivity. *Br J Psychiatry* 1978; **133:** 429-435.

5. Wechsler D. Wechsler Abbreviated Scale of Intelligence (WASI). In: Association AP (ed). APA PsychTests, 1999.

6. Spitzer RL, Kroenke K, Williams JB. Validation and utility of a self-report version of PRIME-MD: the PHQ primary care study. Primary Care Evaluation of Mental Disorders. Patient Health Questionnaire. *Jama* 1999; **282**(18)**:** 1737-1744.

7. Bigdely-Shamlo N, Mullen T, Kothe C, Su KM, Robbins KA. The PREP pipeline: standardized preprocessing for large-scale EEG analysis. *Front Neuroinform* 2015; **9:** 16.

8. Yoon SJ, Elahi LS, Pașca AM, Marton RM, Gordon A, Revah O *et al.* Reliability of human cortical organoid generation. *Nat Methods* 2019; **16**(1)**:** 75-78.

9. Bolger AM, Lohse M, Usadel B. Trimmomatic: a flexible trimmer for Illumina sequence data. *Bioinformatics (Oxford, England)* 2014; **30**(15)**:** 2114-2120.

10. Kim D, Langmead B, Salzberg SL. HISAT: a fast spliced aligner with low memory requirements. *Nat Methods* 2015; **12**(4)**:** 357-360.

11. Liao Y, Smyth GK, Shi W. featureCounts: an efficient general purpose program for assigning sequence reads to genomic features. *Bioinformatics (Oxford, England)* 2014; **30**(7)**:** 923-930.

12. Hu M, Chikina M. InstaPrism: an R package for fast implementation of BayesPrism. *Bioinformatics (Oxford, England)* 2024; **40**(7).

13. Chu T, Wang Z, Pe'er D, Danko CG. Cell type and gene expression deconvolution with BayesPrism enables Bayesian integrative analysis across bulk and single-cell RNA sequencing in oncology. *Nature cancer* 2022; **3**(4)**:** 505-517.

14. Wang L, Wang C, Moriano JA, Chen S, Zuo G, Cebrián-Silla A *et al.* Molecular and cellular dynamics of the developing human neocortex. *Nature* 2025.

15. Akkouh IA, Ueland T, Szabo A, Hughes T, Smeland OB, Andreassen OA *et al.* Longitudinal Transcriptomic Analysis of Human Cortical Spheroids Identifies Axonal Dysregulation in the Prenatal Brain as a Mediator of Genetic Risk for Schizophrenia. *Biol Psychiatry* 2024; **95**(7)**:** 687-698.

16. Osete JR, Akkouh IA, Ievglevskyi O, Vandenberghe M, de Assis DR, Ueland T *et al.* Transcriptional and functional effects of lithium in bipolar disorder iPSC-derived cortical spheroids. *Mol Psychiatry* 2023.

**Supplementary Figures and Tables**

**Supplementary Figure 1.** Estimation of cell type composition.

**Supplementary Figure 2.** Characteristics of cortical spheroid neurons

**Supplementary Figure 3**. Analysis of inward Na^+^ and spontaneous postsynaptic currents (sEPSCs) parameters.

**Supplementary Table 1.** Demographic and clinical details about the study participants.

**Supplementary Table 2.** Primary and secondary antibodies used for IHC.

**Supplementary Table 3.** Analysis of selected neuronal properties in CTRL and SZ hCS neurons.

**Supplementary Table 4.** Parameters of sEPSCs recorded in CTRL and SZ hCS neurons.

**Supplementary Table 5.** Analyses of associations between EEG variables and hCS readouts.

**Supplementary Table 6.** Secondary analyses of associations between EEG variables and hCS readouts.

**Supplementary Figure Legends**

**Supplementary Figure 1. Estimation of cell type composition. A)** UMAP plot of the primary human single-cell reference data used to construct cell type signatures. **B)** Gene expression distribution of the glutamatergic neuron marker *SLC17A7* (*VGLUT1*) and the GABAergic marker *GAD1*. **C)** Estimated cell type fractions per disease group. IPC-EN: Intermediate progenitor cells of the excitatory neuronal lineage. OPC: Oligodendrocyte precursor cells.

**Supplementary Figure 2. Characteristics of cortical spheroid neurons.**

A, B - Representative DIC and corresponding fluorescent images of neurons filled with ALexa Fluor488 during patch clamp recording from each experimental group (CTRL/SZ and CTRL/BD). Scale bar corresponds to 50μm.

C -Comparison of rest membrane potential (averaged) in CTRL vs SZ and CTRL vs BD. t-test comparison p>0.05.

1. Comparison of membrane capacitance (pF) in recorded neurons from CTRL/SZ and CTRL/BD groups. Average values for SZ group as statistically different from CTRL (p<0.005).
2. Graphical plot of average membrane resistance (MOhm) in stated neuron groups. The average value for SZ was significantly different compared to CTRL (p<0.001).
3. Comparison of input resistance values (MOhm) in experimental groups. Values ere significantly lower for SZ group neurons (p<0.005, t test).
4. Graphical representation plots for cell time constant (ms) in all experimental groups. No significance yielded in group comparison.
5. Comparison of access resistance values (MOhm) in CTRL/SZ and CTRL/BD group neurons. The values were significantly lower in BD neurons (p<0.005, t test).

**Supplementary Figure 3. Analysis of inward Na^+^ and spontaneous postsynaptic currents (sEPSCs) parameters.**

A -Comparison of current density at the peak of current for all experimental groups. The values for the SZ group neurons were significantly different compared to the CTRL <0.05, t test).

B -Graphical plots of voltage threshold for Na^+^ current activation.

1. IV curves for sodium currents related to current density (amplitude normalized to capacitance).
2. Examples of recorded sEPSC traces in neuron from the CTRL group (upper panel) and from the SZ group.
3. Comparison of amplitudes (upper panel) and frequencies (lower panel) of sEPSCs recorded from neurons in the CTRL and in the SZ group. No significant differences (p>0.05, t-test) were found.

F -The kinetics of sEPSCs such as rise time (τrise) and decay time (τdecay) were not significantly different in neurons CTRL vs SZ groups. p>0.05, t-test.

**Supplementary Figure 1**


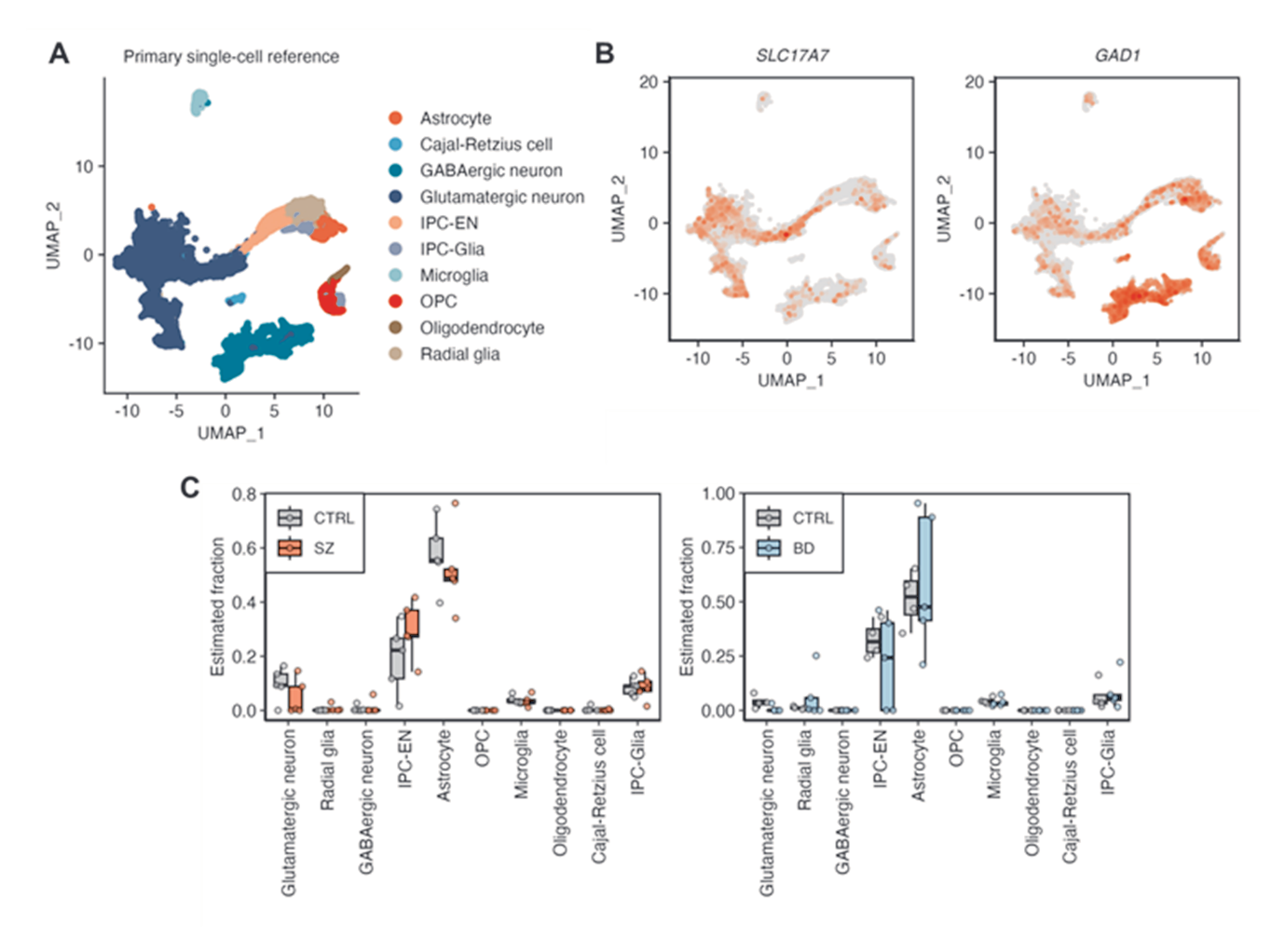


**Supplementary Figure 2**


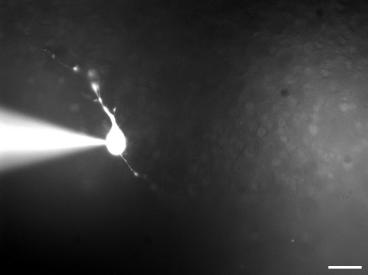

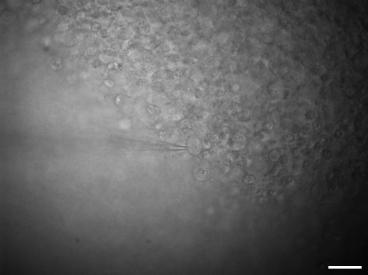

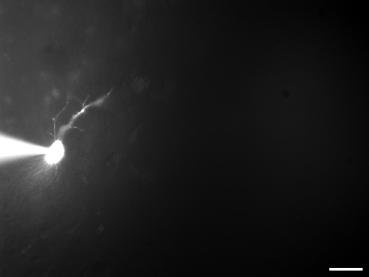

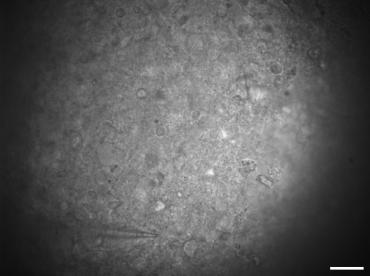

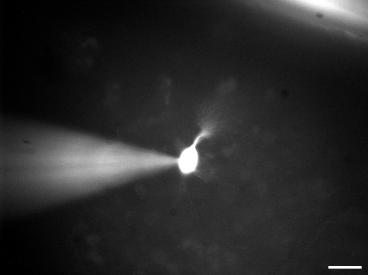

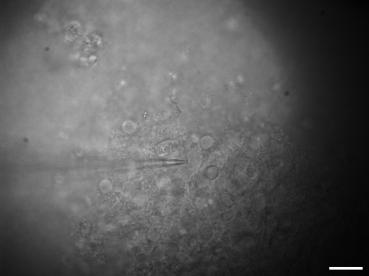

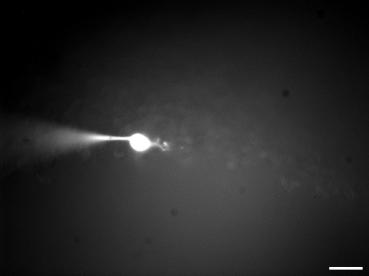

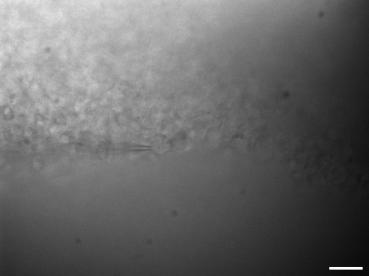

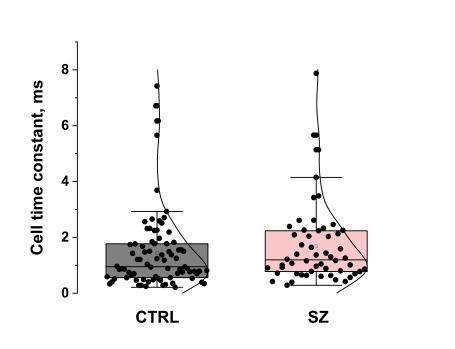

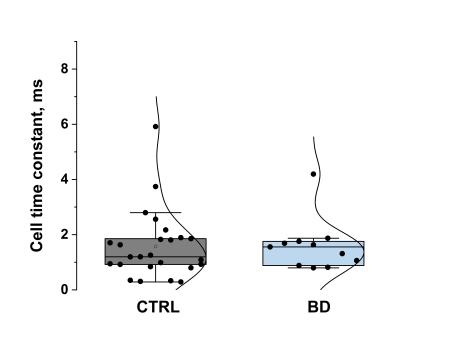

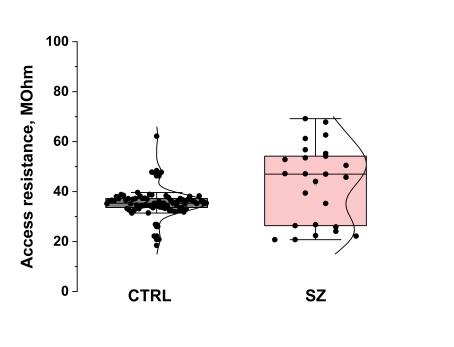

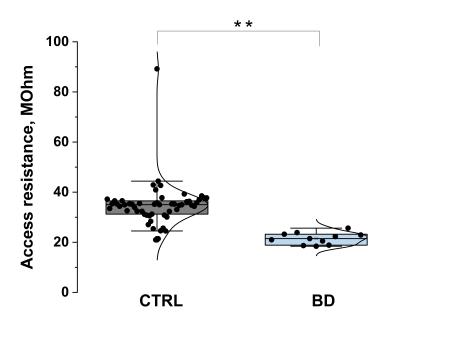


SZ

CTRL

BD


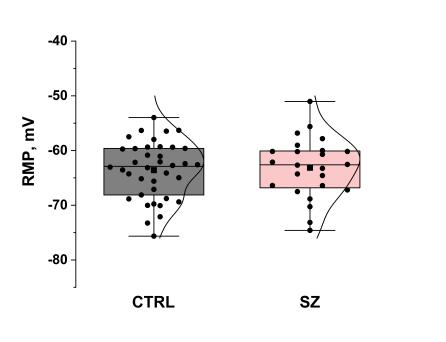

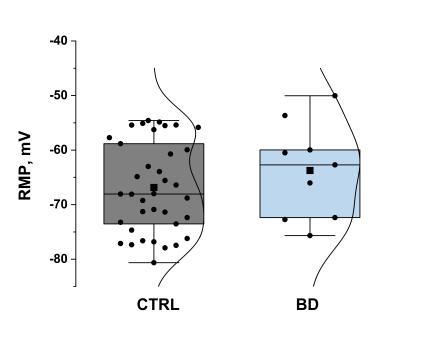

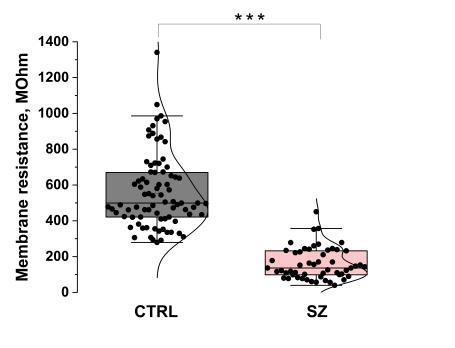


CTRL

**A**

**G**

**D**

**C**

**B**

**E**


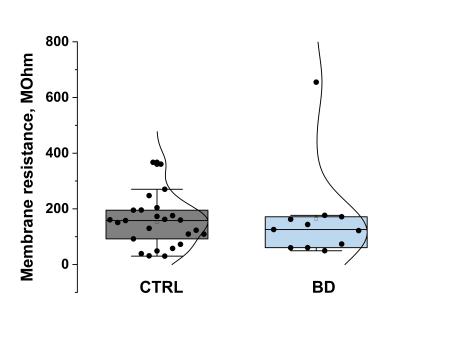


H


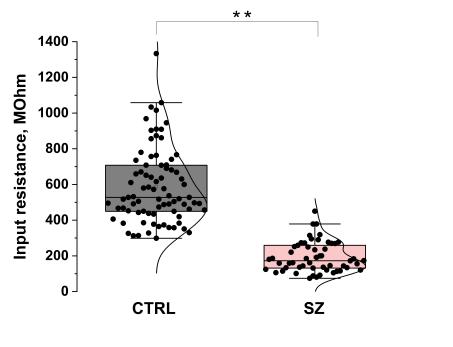

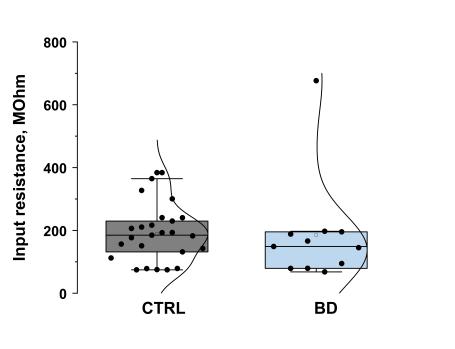

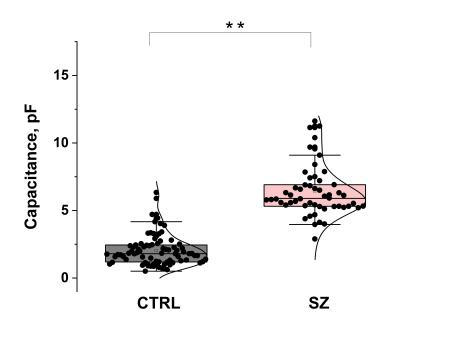


**F**


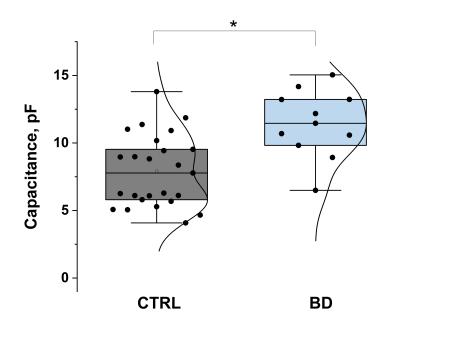


**Supplementary Figure 3**


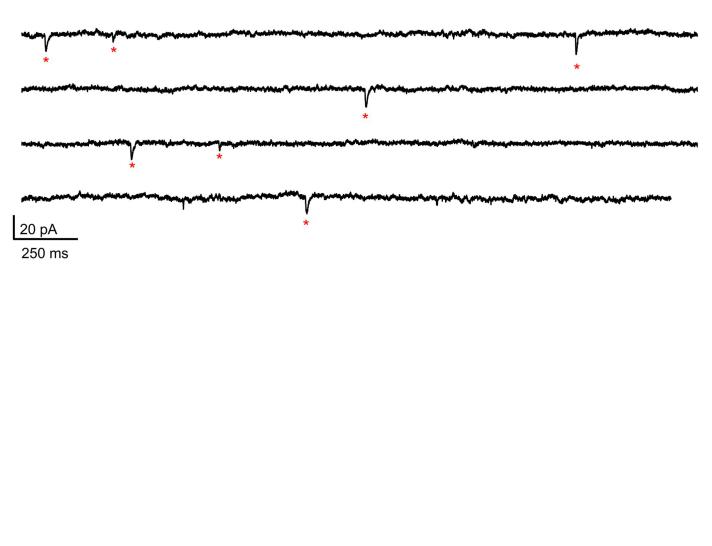

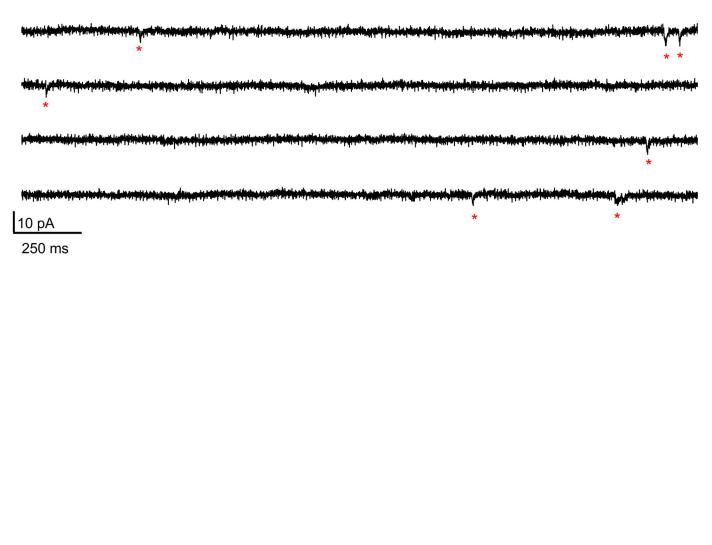

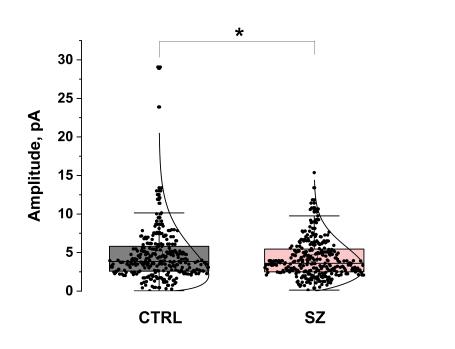

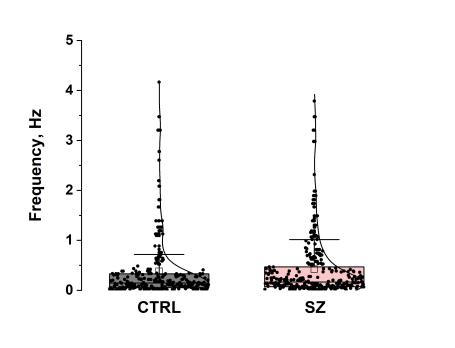

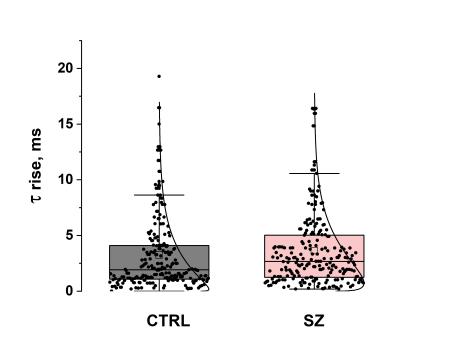

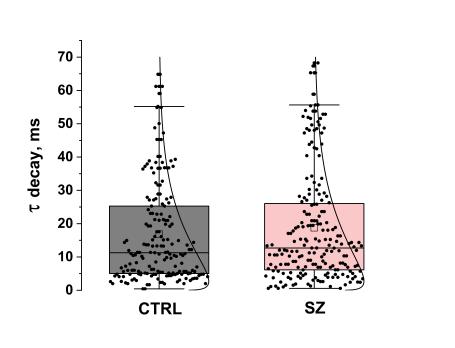


**SZ**

**CTRL**


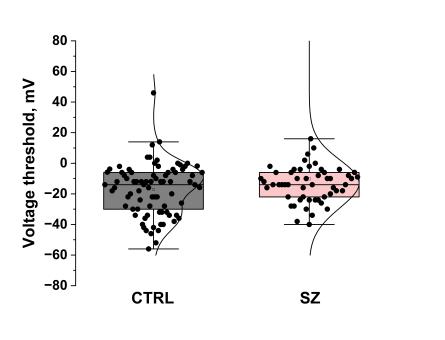

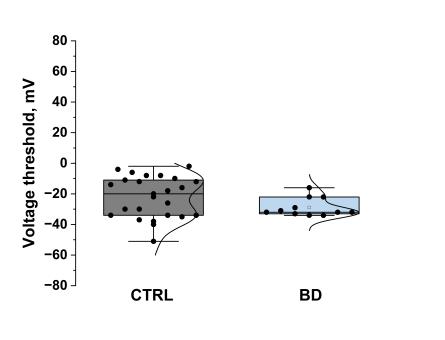

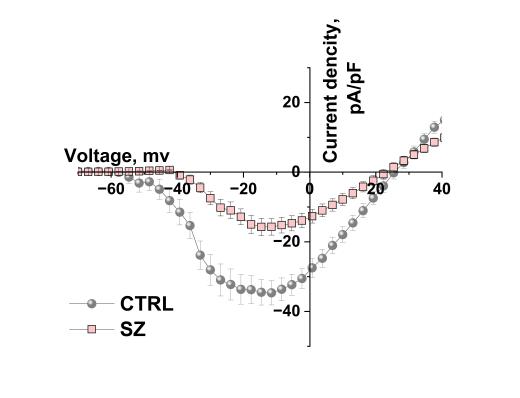

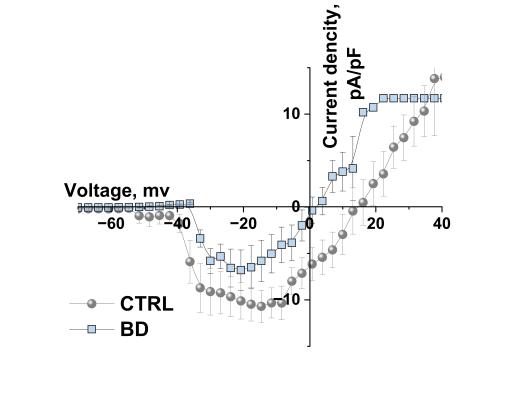


**A**

**B**

**C**

**D**

**E**

**F**


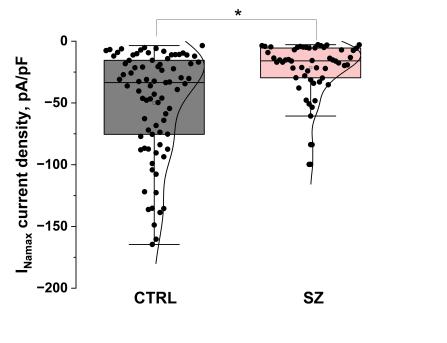

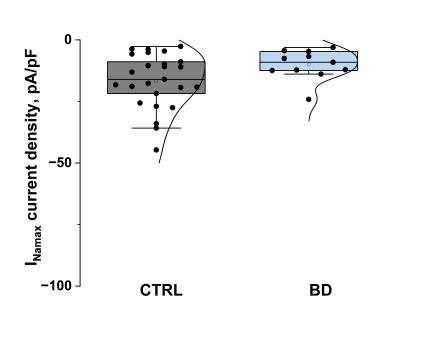


**Supplementary Table 1.** Demographic and clinical details about the study participants.

|  | **CTRL (n=5)** | **BD (n=5)** | **SZ(n=5)** | **p.value** |
| --- | --- | --- | --- | --- |
| **Age (years; mean,sd)** | 45.6 (9.8) | 39.0 (14.2) | 43.8 (12.2) | 0.60 |
| **Females (n, &)** | 2/5 (40%) | 3/5 (60%) | 2/5 (40%) | 0.77 |
| **GAF-F (mean, sd)** | **-** | 59.2 (12.1) | 48.8 (18.2) | 0.32 |
| **IQ (mean, sd)** | 116.0 (4.6) | 111.0 (16.6) | 112.4 (12.0) | 0.80 |
| **PANSS (mean, sd)** | **-** | 44.8 (11.7) | 57.8 (15.7) | 0.18 |
| **MMN (mean, sd)** | -2.28 (0.63) | -2.43 (1.69) | -2.17 (1.28 | 0.95 |
| **LTP (mean, sd)** | -2.12 (3.18) | -1,04 (1.55) | -1.04 (1.15) | 0.70 |

| **Supplementary Table 2.** Primary and secondary antibodies used for IHC**.** | | | |  |
| --- | --- | --- | --- | --- |
|  |  |  |  |  |
| **Antibody** | **Species** | **Dilution** | **Company** | **Reference** |
|  |  |  |  |  |
| Parvalbumin | mouse | 1:1000 | Novus Biologicals | NBP2-50038 |
| Somatostatin | rat | 5 μg/mL | R&D Systems | MAB2358 |
| GRIN2A | rabbit | 1:1000 | Proteintech | 28571-1-AP |
| VGlut1 | rabbit | 1: 500 | Synaptic Systems | 135302 |
| MAP2 | chicken | 1:10 000 | Abcam | ab5392 |
| Anti-mouse Alexa 488 | rabbit | 1:200 | Invitrogen | A11059 |
| Anti-rabbit Alexa 647 | goat | 1:200 | Invitrogen | A32733 |
| Anti-rat Alexa 594 | donkey | 1:200 | Invitrogen | A2109 |
| Anti-chicken Alexa 594 | goat | 1:200 | Invitrogen | A21449 |
| Anti-rabbit Alexa594 | donkey | 1:200 | Invitrogen | A21207 |

**Supplementary Table 3.** Analysis of selected neuronal properties in CTRL and SZ hCS neurons.

|  | CTRL 180 DIV |  | SZ  180 DIV |  | Two sample  t test | CTRL 180 DIV |  | BD  180 DIV |  | Two sample  t test |
| --- | --- | --- | --- | --- | --- | --- | --- | --- | --- | --- |
|  | Mean | SE | Mean | SE | P value | Mean | SE | Mean | SE | P value |
| **Na current max amplitude, pA** | -291.83 | 26.64 | -235.49 | 30.08 | 0.16 | -168.7 | 27.94 | -120.85 | 20.14 | 0.29 |
| **Na current density (at peak current)**  **pA/pA** | -49.88 | 4.76 | -21.3 | 2.74 | 0.00011 | -16.63 | 2.22 | -10.03 | 1.8 | 0.074 |
| **Na current threshold, mV** | -17.13 | 1.88 | -10.11 | 3.90 | 0.076 | -22.07 | 2.69 | -28.84 | 1.82 | 0.12 |
| **RMP, mV** | -63.59 | 0.84 | -63.14 | 1.13 | 0.98 | -69.06 | 2.39 | -60.42 | 2.91 | 0.08 |
| **Capacitance, pF** | 2.04 | 0.13 | 6.43 | 0.26 | 0.001 | 7.9 | 0.53 | 11.4 | 0.75 | 0.006 |
| **Membrane resistance, MOhM** | 561.67 | 23.18 | 159.95 | 11.92 | 0.00022 | 152.99 | 18.12 | 163.77 | 51.14 | 0.8 |
| **Input resistance, MOhM** | 587.57 | 23.12 | 193.31 | 11.47 | 0.00058 | 189 | 17.63 | 185.29 | 51.29 | 0.93 |
| **Access resistance, MOhM** | 35.58 | 0.65 | 34.82 | 1.22 | 0.56 | 43.16 | 3.14 | 21.53 | 0.7 | 0.0007 |
| **Cell time constant, ms** | 1.4 | 0.15 | 1.68 | 0.19 | 0.34 | 1.57 | 0.25 | 1.6 | 0.29 | 0.955 |
| **I threshold, pA** | 27.58 | 2.67 | 31.54 | 4.6 | 0.86 | 11.95 | 2.28 | 57.05 | 14.35 | 0.036 |

**Supplementary Table 4.** Parameters of sEPSC recorded in CTRL and SZ hCS neurons

|  | CTRL 180 DIV |  | SCZ 180 DIV |  | Two sample  t test |
| --- | --- | --- | --- | --- | --- |
|  | Mean | SE | Mean | SE | P value |
| Peak amplitude, pA | 4.82 | 0.23 | 4.27 | 0.14 | 0.041 |
| tau_rise, ms | 5.13 | 0.7 | 5.15 | 0.52 | 0.98 |
| tau_decay, ms | 20.59 | 1.52 | 23.25 | 1.58 | 0.76 |
| Frequency, Hz | 0.38 | 0.04 | 0.43 | 0.04 | 0.342 |

**Supplementary Table 5.** Associations between EEG variables and hCS readouts.

| \| **Associations between EEG variables and hCS readouts** \| \| \| \| \| \| \| \| \| \| --- \| --- \| --- \| --- \| --- \| --- \| --- \| --- \| --- \| \|  \|  \|  \| **Stepwise regression: LTP~hCS variable + diagnosis + batch + age + sex** \| \| \| \| \| \| \|  \|  \|  \| **AIC** \| **R2-adj** \| **t.value** \| **st.β** \| **p.value*** \| **Final model** \| \| **I-treshold, pA** \| \| \| 9.65 \| 0.55 \| -1.58 \| -0.56 \| 0.17 \| I-treshold + diagnosis + sex \| \| **Basal respiration** \| \| \| 2.04 \| 0.67 \| -2.60 \| -0.45 \| **0.028** \| Basal resp + sex \| \| **VGlut1** \| \| \| 6.40 \| 0.64 \| -1.99 \| -0.38 \| 0.082 \| VGLUT1 + sex \| \| * All p. values are uncorrected  Ɨ The variable did not make it to the winning model  Abbreviations: EEG, Electroencephalography; hCS, human cortical spheroids; MMN, Mismatch negativity; LTP, Long-term potentiation like plasticity; I-treshold; Input-treshold; ATP, Adenosine triphosphate; VGLUT1, Vesicular glutamate transporter type 1   \|  \| \| --- \| \| \| \| \| \| \| \| \| \| \| \|  \| \|  \| \|  \| \|  \| \|  \| |
| --- | --- | --- | --- | --- | --- | --- | --- | --- | --- | --- | --- | --- | --- | --- | --- | --- | --- | --- | --- | --- | --- | --- | --- | --- | --- | --- | --- | --- | --- | --- | --- | --- | --- | --- | --- | --- | --- | --- | --- | --- | --- | --- | --- | --- | --- | --- | --- | --- | --- | --- | --- | --- | --- | --- | --- | --- | --- | --- | --- | --- | --- | --- | --- | --- | --- | --- | --- | --- | --- |

**Supplementary Table 6.** Secondary association analysis between clinical parameters and hCS read-outs.

| \| **Stepwise regression results clinical variables vs hCS read-outs** \| \| \| \| \| \| \| \| \| \| \| \| --- \| --- \| --- \| --- \| --- \| --- \| --- \| --- \| --- \| --- \| --- \| \| **MMN** \| \| \| \| \| \| \| \| \| \| \| \| **MMN: Initial model; lm(MMN~hCS read-out + batch + diagnosis + age + sex)** \| \| \| \| \| \| \| \| \| \| \| \|  \|  \|  \| **AIC** \| **R2-adj** \| **t-value** \| **p.value*** \| **Final model** \| \| \|  \| \| **Na current max amplitude, pA** \| \| \| -0.35 \| 0.44 \| Ɨ \| \| age + sex \| \| \|  \| \| **Voltage current max, mV** \| \| \| -0.35 \| 0.44 \| Ɨ \| \| age + sex \| \| \|  \| \| **RMP, mV** \| \| \| -0.35 \| 0.44 \| Ɨ \| \| age + sex \| \| \|  \| \| **I-treshold, pA** \| \| \| -0.35 \| 0.44 \| Ɨ \| \| age + sex \| \| \|  \| \| **Basal respiration** \| \| \| 0.03 \| 0.43 \| Ɨ \| \| age + sex \| \| \|  \| \| **ATP respiration** \| \| \| 1.70 \| 0.43 \| 1.53 \| 0.16 \| ATP resp + diagnosis + age + sex \| \| \|  \| \| **Reserve capacity** \| \| \| -2.39 \| 0.19 \| -1.88 \| 0.11 \| reserve capacity + age + batch + diagnosis \| \| \| \| \| **Basal glycolysis** \| \| \| -3.06 \| 0.16 \| Ɨ \| \| age \| \| \|  \| \| **VGlut1** \| \| \| -1.27 \| 0.45 \| Ɨ \| \| age + sex \| \| \|  \| \|  \| \| \|  \|  \|  \| \|  \| \| \|  \| \|  \|  \|  \|  \|  \|  \|  \|  \|  \|  \|  \| \| **LTP-like plasticity** \| \| \| \| \| \| \| \| \| \| \| \| **LTP-like plasticity: Initial model; lm(LTP~hCS read-out + batch + diagnosis + age + sex** \| \| \| \| \| \| \| \| \| \| \| \|  \|  \|  \| **AIC** \| **R2-adj** \| **t.value** \| **p.value*** \| **Final model** \| \| \|  \| \| **Na current max amplitude, pA** \| \| \| 10.40 \| 0.52 \| Ɨ \| \| diagnosis + batch + sex \| \| \|  \| \| **Voltage current max, mV** \| \| \| 10.17 \| 0.53 \| -1.06 \| 0.34 \| Volt curren + diagnosis + batch + sex \| \| \| \| \| **RMP, mV** \| \| \| 8.64 \| 0.56 \| Ɨ \| \| age + sex \| \| \|  \| \| **ATP respiration** \| \| \| 2.97 \| 0.69 \| -2.74 \| **0.022** \| ATP-resp + sex \| \| \|  \| \| **Reserve capacity** \| \| \| 6.08 \| 0.61 \| Ɨ \| \| diagnosis + age + sex \| \| \|  \| \| **Basal glycolysis** \| \| \| 10.88 \| 0.83 \| -3.41 \| 0.18 \| Basal glyc + diagnosis + sex + batch \| \| \| \| \|  \| \| \|  \|  \|  \|  \|  \| \| \|  \| \|  \|  \|  \|  \|  \|  \|  \|  \|  \|  \|  \| \| **PANSS** \| \| \| \| \| \| \| \| \| \| \| \| **PANSS: Initial model; lm(PANSS total score ~ hCS read-out + batch + diagnosis + age + sex)** \| \| \| \| \| \| \| \| \| \| \| \|  \|  \|  \| **AIC** \| **R2-adj** \| **t-value** \| **p.value*** \| **Final model** \| \| \|  \| \| **Na current max amplitude, pA** \| \| \| 45.79 \| 0.09 \| Ɨ \| \| diagnosis \| \| \|  \| \| **Voltage current max, mV** \| \| \| 45.79 \| 0.09 \| Ɨ \| \| diagnosis \| \| \|  \| \| **RMP, mV** \| \| \| 42.52 \| 0.40 \| -2.38 \| 0.055 \| RMP \| \| \|  \| \| **I-treshold, pA** \| \| \| 45.79 \| 0.09 \| Ɨ \| \| diagnosis \| \| \|  \| \| **Basal respiration** \| \| \| 50.74 \| 0.45 \| 2.42 \| 0.052 \| Basal rep + age + sex \| \| \|  \| \| **ATP respiration** \| \| \| 49.42 \| 0.49 \| 2.72 \| **0.03** \| ATP resp + age \| \| \|  \| \| **Reserve capacity** \| \| \| 44.41 \| 0.15 \| -1.62 \| 0.18 \| Res.capacity + diagnosis + age \| \| \|  \| \| **Basal glycolysis** \| \| \| 48.04 \| 0.21 \| Ɨ \| \| diagnosis + sex \| \| \|  \| \| **VGlut1** \| \| \| 46.75 \| 0.63 \| 3.42 \| **0.014** \| VGLUT1 + age + sex \| \| \|  \| \|  \| \| \|  \|  \|  \| \|  \| \| \|  \| \|  \| \| \| \| \| \| \| \| \| \| \| \| **IQ** \| \| \| \| \| \| \| \| \| \| \| \| **IQ: Initial model; lm(IQ (WASI) ~ hCS read-out + batch + diagnosis + age + sex)** \| \| \| \| \| \| \| \| \| \| \| \|  \|  \|  \| **AIC** \| **R2-adj** \| **t-value** \| **p.value*** \| **Final model** \| \| \|  \| \| **Na current max amplitude, pA** \| \| \| 48.29 \| 0.50 \| -1.97 \| 0.077 \| Na curr max amp + diagnosis \| \| \|  \| \| **Voltage current max, mV** \| \| \| 41.08 \| 0.71 \| -3.77 \| **0.004** \| Voltage current + sex \| \| \|  \| \| **RMP, mV** \| \| \| 50.56 \| 0.37 \| Ɨ \| \| sex \| \| \|  \| \| **I-treshold, pA** \| \| \| 50.56 \| 0.37 \| Ɨ \| \| sex \| \| \|  \| \| **Basal respiration** \| \| \| 70.15 \|  \| Ɨ \| \| No variables entered final model \| \| \|  \| \| **ATP respiration** \| \| \| 70.15 \|  \| Ɨ \| \| No variables entered final model \| \| \|  \| \| **Reserve capacity** \| \| \| 60.31 \| 0.16 \| -1.50 \| 0.18 \| Res.cap + diagnosis + sex \| \| \|  \| \| **Basal glycolysis** \| \| \| 60.76 \| 0.10 \| 1.50 \| 0.16 \| Basal glycolysis \| \| \|  \| \| **VGlut1** \| \| \| 50.46 \| 0.37 \| Ɨ \| \| sex \| \| \|  \| \|  \| \| \|  \|  \|  \|  \|  \| \| \|  \| \|  \| \| \| \| \| \| \| \| \| \| \| \| **GAF-F** \| \| \| \| \| \| \| \| \| \| \| \| **GAF-F: Initial model; lm(GAF-F~ hCS read-out + batch + diagnosis + age + sex)** \| \| \| \| \| \| \| \| \| \| \| \|  \|  \|  \| **AIC** \| **R2-adj** \| **t-value** \| **p.value*** \| **Final model** \| \| \|  \| \| **Na current max amplitude, pA** \| \| \| 44.18 \| 0.36 \| Ɨ \| \| age + sex \| \| \|  \| \| **Voltage current max, mV** \| \| \| 44.18 \| 0.36 \| Ɨ \| \| age + sex \| \| \|  \| \| **RMP, mV** \| \| \| 44.18 \| 0.36 \| Ɨ \| \| age + sex \| \| \|  \| \| **I-treshold, pA** \| \| \| 44.18 \| 0.36 \| Ɨ \| \| age + sex \| \| \|  \| \| **Basal respiration** \| \| \| 55.93 \| 0.17 \| -1.20 \| 0.28 \| Basal resp + age + sex \| \| \|  \| \| **ATP respiration** \| \| \| 54.63 \| 0.24 \| -1.59 \| 0.16 \| ATP resp + age \| \| \|  \| \| **Reserve capacity** \| \| \| 46.75 \| 0.17 \| 1.34 \| 0.25 \| Res. capacity +diagnosis + age \| \| \|  \| \| **Basal glycolysis** \| \| \| 51.21 \| 0.10 \| Ɨ \| \| age \| \| \|  \| \| **VGlut1** \| \| \| 42.23 \| 0.50 \| -1.68 \| 0.19 \| VGlut1 + diagnosis + age + sex \| \| \|  \| \|  \| \| \|  \|  \|  \| \|  \| \| \|  \| \| \|  \| \| --- \| \| \| \| \| \| \| \| \| \| \| \| \| \|  \| \|  \| \|  \| \|  \| \|  \| \|  \|   * All p.values are uncorrected Ɨ The variable did not enter into the final model  Abbreviations: EEG, Electroencephalography; hCS, human cortical spheroids; PANSS, Positive and negative syndrome scale; VEP, IQ, Intelligence quotient; GAF-F, Global assessment of functioning- functioning subscale; St.β, Standardized beta coefficient; Na, Sodium; RMP, Resting membrane potential; I-treshold; ATP, Adenine triphosphate; VGlut1, Vesicular glutamate transporter 1; MAP2, Microtubule associated protein; PV, Parvalbumin; GRIN2A, Glutamate receptor ionotropic type NMDA subunit 2A |
| --- | --- | --- | --- | --- | --- | --- | --- | --- | --- | --- | --- | --- | --- | --- | --- | --- | --- | --- | --- | --- | --- | --- | --- | --- | --- | --- | --- | --- | --- | --- | --- | --- | --- | --- | --- | --- | --- | --- | --- | --- | --- | --- | --- | --- | --- | --- | --- | --- | --- | --- | --- | --- | --- | --- | --- | --- | --- | --- | --- | --- | --- | --- | --- | --- | --- | --- | --- | --- | --- | --- | --- | --- | --- | --- | --- | --- | --- | --- | --- | --- | --- | --- | --- | --- | --- | --- | --- | --- | --- | --- | --- | --- | --- | --- | --- | --- | --- | --- | --- | --- | --- | --- | --- | --- | --- | --- | --- | --- | --- | --- | --- | --- | --- | --- | --- | --- | --- | --- | --- | --- | --- | --- | --- | --- | --- | --- | --- | --- | --- | --- | --- | --- | --- | --- | --- | --- | --- | --- | --- | --- | --- | --- | --- | --- | --- | --- | --- | --- | --- | --- | --- | --- | --- | --- | --- | --- | --- | --- | --- | --- | --- | --- | --- | --- | --- | --- | --- | --- | --- | --- | --- | --- | --- | --- | --- | --- | --- | --- | --- | --- | --- | --- | --- | --- | --- | --- | --- | --- | --- | --- | --- | --- | --- | --- | --- | --- | --- | --- | --- | --- | --- | --- | --- | --- | --- | --- | --- | --- | --- | --- | --- | --- | --- | --- | --- | --- | --- | --- | --- | --- | --- | --- | --- | --- | --- | --- | --- | --- | --- | --- | --- | --- | --- | --- | --- | --- | --- | --- | --- | --- | --- | --- | --- | --- | --- | --- | --- | --- | --- | --- | --- | --- | --- | --- | --- | --- | --- | --- | --- | --- | --- | --- | --- | --- | --- | --- | --- | --- | --- | --- | --- | --- | --- | --- | --- | --- | --- | --- | --- | --- | --- | --- | --- | --- | --- | --- | --- | --- | --- | --- | --- | --- | --- | --- | --- | --- | --- | --- | --- | --- | --- | --- | --- | --- | --- | --- | --- | --- | --- | --- | --- | --- | --- | --- | --- | --- | --- | --- | --- | --- | --- | --- | --- | --- | --- | --- | --- | --- | --- | --- | --- | --- | --- | --- | --- | --- | --- | --- | --- | --- | --- | --- | --- | --- | --- | --- | --- | --- | --- | --- | --- | --- | --- | --- | --- | --- | --- | --- | --- | --- | --- | --- | --- | --- | --- | --- | --- | --- | --- | --- | --- | --- | --- | --- | --- | --- | --- | --- | --- | --- | --- | --- | --- | --- | --- | --- | --- | --- | --- | --- | --- | --- | --- | --- | --- | --- | --- | --- | --- | --- | --- | --- | --- | --- | --- | --- | --- | --- | --- | --- | --- | --- | --- | --- | --- | --- | --- | --- | --- | --- | --- | --- | --- | --- | --- | --- | --- | --- | --- | --- | --- | --- | --- | --- | --- | --- | --- | --- | --- | --- | --- | --- | --- | --- | --- | --- | --- | --- | --- | --- | --- | --- | --- | --- | --- | --- | --- | --- | --- | --- | --- | --- | --- | --- | --- | --- | --- | --- | --- | --- | --- | --- | --- | --- | --- | --- | --- | --- | --- | --- | --- | --- | --- | --- | --- | --- | --- | --- | --- | --- | --- | --- | --- | --- | --- | --- | --- | --- | --- | --- | --- | --- | --- | --- | --- | --- | --- | --- | --- | --- | --- | --- | --- | --- | --- | --- | --- | --- | --- | --- | --- | --- | --- | --- | --- | --- | --- | --- | --- | --- | --- | --- | --- | --- | --- | --- | --- | --- | --- | --- | --- | --- | --- | --- | --- | --- | --- | --- | --- | --- | --- | --- | --- | --- | --- | --- | --- | --- | --- | --- | --- | --- | --- | --- | --- | --- | --- | --- | --- | --- | --- | --- | --- | --- | --- | --- | --- | --- | --- | --- | --- | --- | --- | --- | --- | --- | --- | --- | --- | --- | --- | --- | --- | --- | --- | --- | --- | --- | --- | --- | --- | --- | --- | --- | --- | --- | --- | --- | --- | --- | --- | --- | --- | --- | --- | --- | --- | --- | --- | --- | --- | --- | --- | --- | --- | --- | --- | --- | --- | --- | --- | --- | --- | --- | --- | --- | --- | --- | --- | --- | --- | --- | --- | --- | --- | --- | --- | --- | --- | --- | --- | --- | --- | --- | --- | --- | --- | --- | --- | --- | --- | --- | --- | --- | --- | --- | --- | --- | --- | --- | --- | --- | --- | --- | --- | --- | --- | --- | --- | --- | --- | --- | --- | --- | --- | --- | --- | --- | --- | --- | --- | --- | --- | --- | --- | --- | --- | --- | --- | --- | --- | --- | --- | --- | --- | --- | --- | --- | --- | --- | --- | --- | --- | --- | --- | --- | --- | --- | --- | --- | --- | --- | --- | --- | --- | --- | --- | --- | --- | --- | --- | --- | --- | --- | --- | --- | --- | --- | --- | --- | --- | --- | --- | --- | --- | --- | --- | --- | --- | --- | --- | --- | --- | --- | --- |
