# Supplementary figures and images for: Investigating neural impairments in psychotic disorders using electroencephalography and cortical spheroids
Source: Transl Psychiatry. 2026 Feb 17;16:114. doi: 10.1038/s41398-026-03863-4 (PMC12949052; doi:10.1038/s41398-026-03863-4)

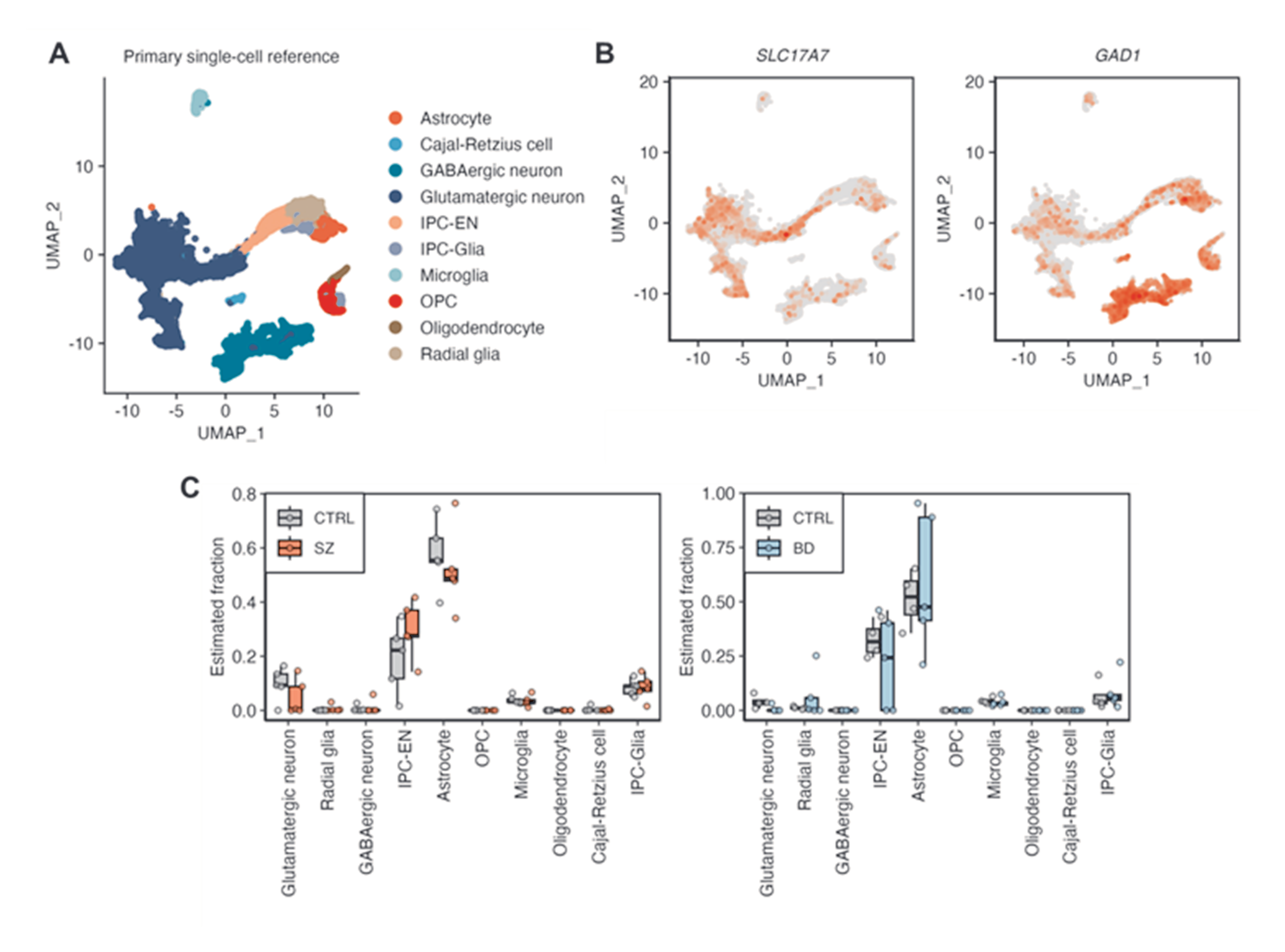

Supplement: Supplementary file 2 — Supplementary Figure 1 [file 41398_2026_3863_MOESM2_ESM.png]

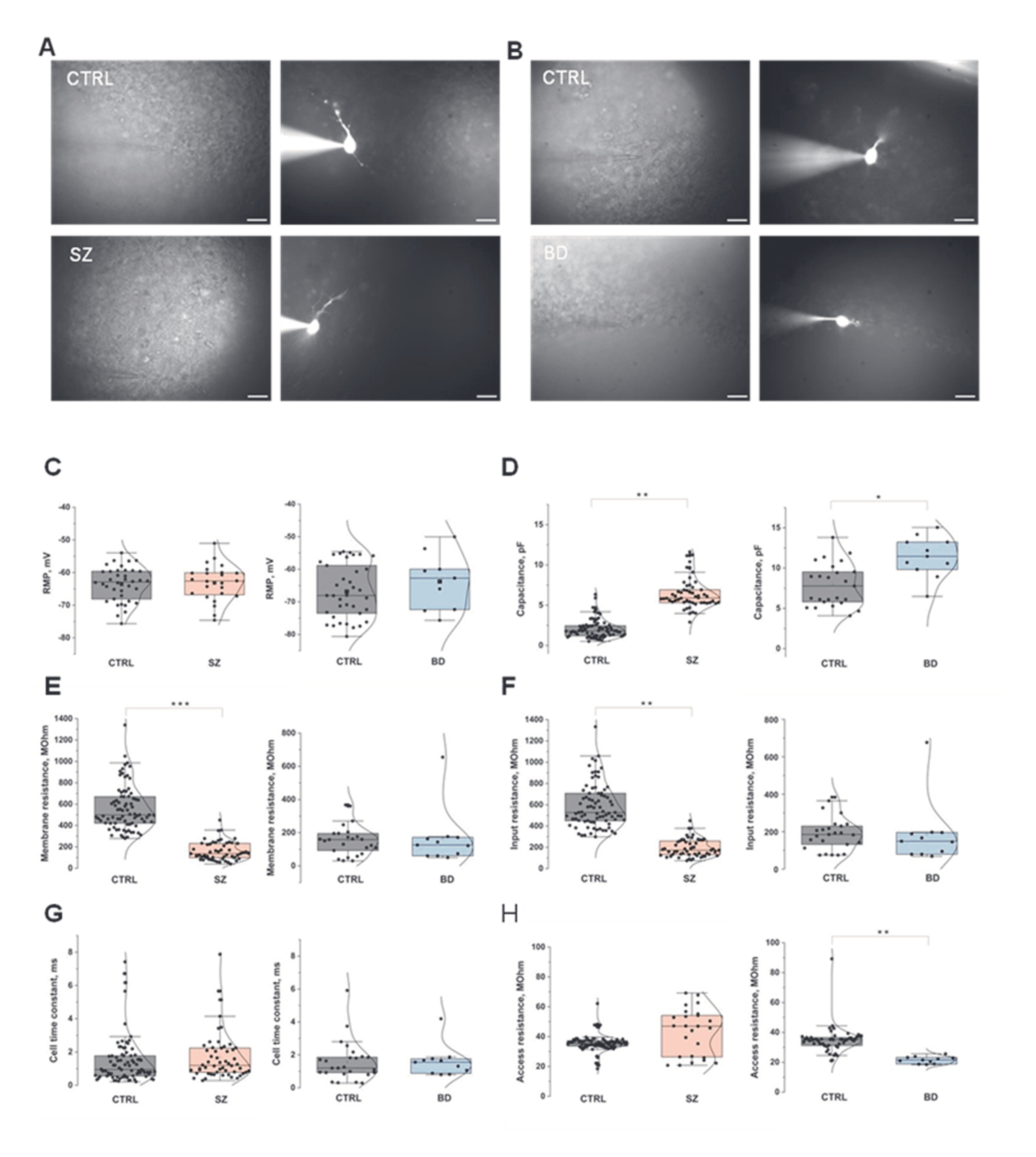

Supplement: Supplementary file 3 — Supplementary Figure 2 [file 41398_2026_3863_MOESM3_ESM.png]

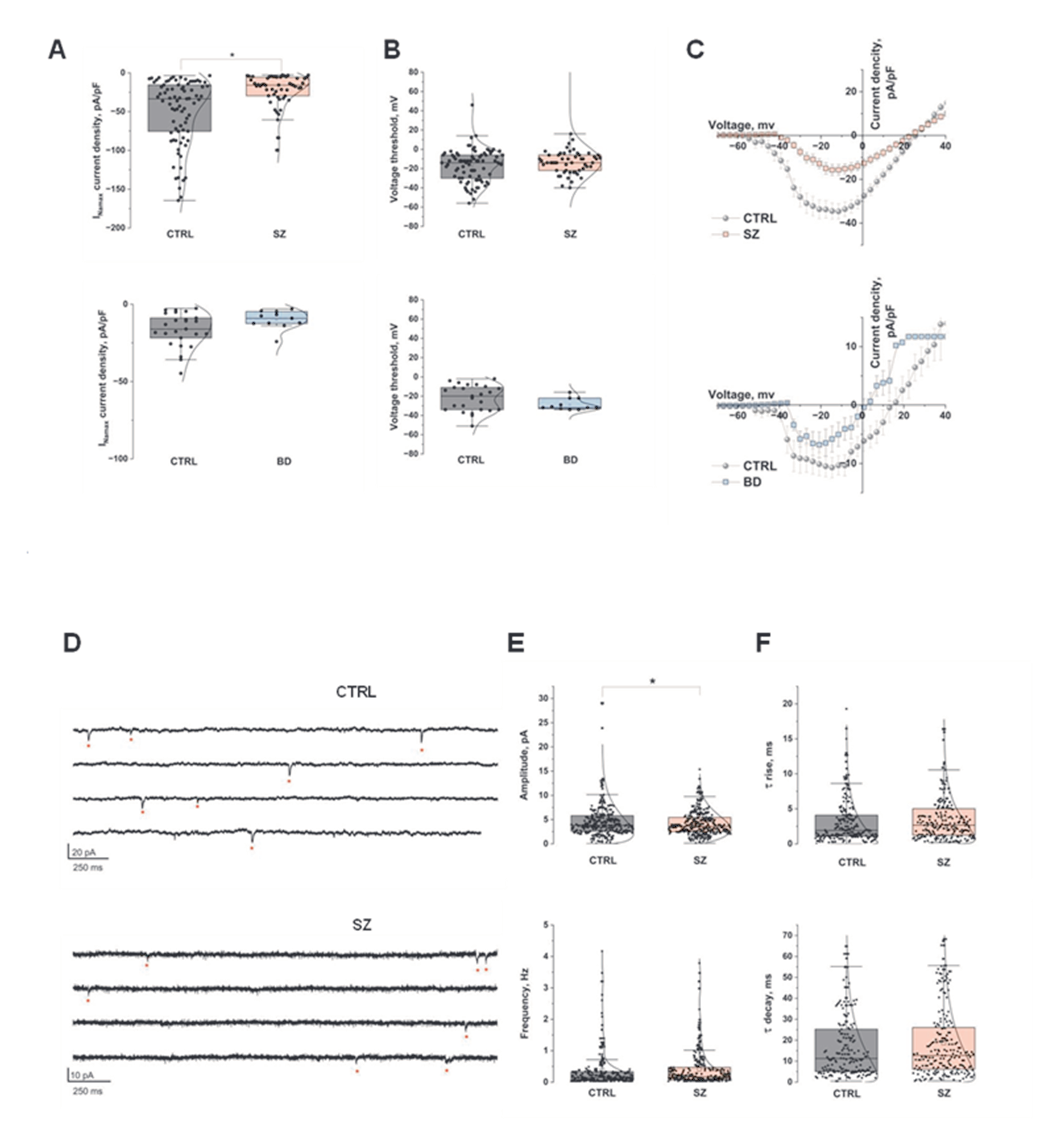

Supplement: Supplementary file 4 — Supplementary Figure 3 [file 41398_2026_3863_MOESM4_ESM.png]
